# Transcriptome Profile of Human Fibroblasts in an *Ex Vivo* Culture

**DOI:** 10.7150/ijms.35693

**Published:** 2020-01-01

**Authors:** Bogusław Machaliński, Dorota Rogińska, Kamila Szumilas, Alicja Zawiślak, Aleksandra Wilk, Iwona Stecewicz, Andrzej Brodkiewicz, Barbara Wiszniewska

**Affiliations:** 1 Department of General Pathology, Pomeranian Medical University, Powstańców Wlkp. 72, 70-111 Szczecin, Poland; 2 Department of Histology and Embryology, Pomeranian Medical University, Powstańców Wlkp. 72, 70-111 Szczecin, Poland

**Keywords:** autologous fibroblasts culture, transcriptome profile, gene expression

## Abstract

Implantation of autologous fibroblasts is a method used to correct age-related changes in facial skin. The aim of this study was to establish the optimal population of cultured human fibroblasts according to the organization of the extracellular matrix in the dermis. Transcriptome profile analysis of cells derived from three consecutive passages indicated that fibroblasts after the second passage were the population with the greatest number of upregulated genes encoding the critical biological processes responsible for skin regeneration, such as extracellular matrix organization, collagen fibril organization, and cell adhesion. Furthermore, genes encoding proteinases responsible for the degradation of dermal extracellular matrix proteins were noticeably downregulated at this stage of culture. Autologous fibroblasts seem to be an optimal and safe biological filler for the renewal of all skin structures.

## Introduction

Skin aging in male and female subjects is a part of natural human aging of different organs, tissues and cells 1. Aging is a complex, inevitable process influenced by a combination of endogenous and exogenous factors. Both types of factors induce cumulative morphological and physiological alterations and progressive changes in each layer of skin as well as influence the appearance of skin 1-3. However, the most pronounced changes occur in elements of the dermis 2, 4, which predominantly consists of connective tissue. During the aging process, human skin undergoes structural and molecular alterations, especially at the level of type I collagen, which is the principle structural component of the dermis 5. These modifications can include rearrangements and reorientation of collagen fibers and increases in the density of collagen bundles. As a consequence, the dermis becomes fragmented, disorganized, and less soluble 5-7. Matrix metalloproteinases (MMPs) are upregulated and collagen synthesis is reduced during skin aging, disrupting the balance between synthesis and degradation processes 7, 8. The decrease in collagen type I disturbs the collagen type III to collagen type I ratio 7, 9. Human skin is rich in elastic fibers. However, the relative volume and quantity of the fibers varies with age, and numerous age-related changes are observed 7, 10-12. A large amount of disintegrated elastic fibers have been reported in individuals older than 70 years 10, 13. The remodeling and degradation of elastic fibers are mostly regulated by MMPs 14, 15, and abnormal localization of elastin in the upper dermis is observed, especially in photo-damaged skin 16. In addition to collagen and elastin, glycosaminoglycans (GAGs) are abundant structural components of the extracellular matrix of human skin. Glycosaminoglycans have various structural and physiological regulatory functions in skin, including tissue water maintenance, given their high water-holding capacity. The levels and localization of GAGs change during endogenous-induced aging to some extent, especially during photoaging 17. The differences in the location of dermal GAGs in photoaged skin as well as alterations in the size, structure and type of GAGs present in sun-damaged skin may affect their water binding properties and their ability to interact with other components of the dermal extracellular matrix 18. Although the total content of GAGs is increased in aged skin, they are unable to bind water. Therefore, poor hydration and a low turgor capacity contributes to the dry (xerotic) appearance of skin 7, 19.

During aging, fibroblasts also play a key role in maintaining the homeostasis of the extracellular matrix and are subjected to various changes, such as reduced metabolic activity, e.g., loss of type I collagen expression 20, 21. Human fibroblasts located within the age-associated dermal extracellular matrix microenvironment express increased levels of collagen-degrading matrix metalloproteinases (MMPs) and decreased levels of tissue inhibitors of MMPs 14, 15, 22.

Due to structural and functional changes, the skin shows obvious signs of the passing time 1. The most important and prominent sign of aging is skin wrinkling 23. Ongoing efforts for preserving a youthful appearance demonstrate that youthfulness is considered a prerequisite for beauty 4. Therefore, the maintenance and improvement of facial skin quality have gained particular attention, and novel technologies aimed at preventing skin aging in women and, more recently, in men are being extensively investigated in the field of aesthetic dermatology. One of the innovative methods used to correct age-related skin changes, e.g., unwanted facial wrinkles, involves the use of cultured fibroblasts derived from the patient`s own skin 24. In our previous studies 24, we observed a significant increase in the diameter of collagen fiber bundles and an improvement in the density of reticular fibers, fibrillin-1-rich microfibrils, and elastic fibers after reinjection of autologous fibroblasts into the dermis.

The aim of this study was to compare global gene expression profiles in fibroblasts from three consecutive passages to estimate the optimal population quality of the tested cells according to potential secretory activity critical for skin rejuvenation.

## Materials and Methods

### Patients and cell culture

Human skin samples were obtained from volunteer male donor at the age of 46, in accordance with the Declaration of Helsinki and with the approval of Local Ethics Committee of Pomeranian Medical University. Skin biopsies (0.8 cm x 0.6 cm) were taken from the postauricular area, which is one of the lower exposed to ultraviolet radiation areas of the head. The skin biopsies were devoted to isolation and culture of dermal fibroblasts and to prepare histological slides.

The skin specimens were transported to the laboratory in ice-cold Ca^2+^/ Mg^2+^-free PBS containing 1:100 penicillin/streptomycin solution and 1 µg/mL Fungizone (both from Thermo Fisher Scientific, Waltham, MA, USA) and then processed immediately. The tissue sample was washed twice with cold Ca^2+^/ Mg^2+^-free PBS, cut into smaller pieces and incubated in 0.6 U/mL Dispase II (Thermo Fisher Scientific, Waltham, MA, USA) for 1-2 h at 37˚C. The epidermis was manually removed from each tissue sample, and the dermis was cut into 1-mm^3^ pieces following enzymatic disaggregation with 0.62 Wünsch U/mL Liberase DH (Roche Applied Science, Penzberg, Germany) for 30-40 min at 37˚C. Subsequently, tissue pieces were dissociated by vortexing and then passed through a 70-µm cell strainer Becton Dickinson, Franklin Lakes, NJ, USA). The dissociated cells were centrifuged at 1500 rpm for 5 minutes. The supernatant was discarded, and the pellet was suspended in Medium 199 (Thermo Fisher Scientific, Waltham, MA, USA) containing 10% human serum isolated from the patient themselves and 0.5% penicillin and streptomycin solution. To obtain human autologous serum, 10 mL of whole blood from patient was collected into plastic tubes containing a serum separator gel with clot activator (Becton Dickinson, Franklin Lakes, NJ, USA). Serum separation was completed after centrifugation at 2,000 rpm for 10 minutes. The cells were cultured in a T75 tissue culture flask (Becton Dickinson, Franklin Lakes, NJ, USA) at 37°C in 5% CO_2_ in a humidified atmosphere. The medium was changed 48-h after plating and every 3-4 days thereafter. When the cultures reached 80% confluency, the cells were detached with Accutase (GE Healthcare, Chicago, IL, USA), washed with PBS and divided in two equal parts. Some of the cells were used to isolate total RNA for microarray gene expression analysis and the remaining part was reseeded in complete medium. The cell cultures were maintained until the 3^rd^ passage.

The samples of skin were fixed in freshly prepared 4% paraformaldehyde and embedded in paraffin. For the morphological analysis, serial slices (3-5 μm in thickness) of skin were mounted onto glass slides to histochemical and immunohistochemical studies.

### RNA isolation

Total RNA from fibroblast cell cultures was isolated using the MirVana Kit (Thermo Fisher Scientific, Waltham, MA, USA), according to the manufacturer's protocol, to create four samples for subsequent microarray analysis: primary fibroblast culture generated from 46-year-old patient (C0) and from 1^st^ (C1), 2^nd^ (C2) and 3^rd^ (C3) passages of cells.

### Affymetrix GeneChip Microarray and Data Analysis

A sense-strand cDNA generated from the total RNA was subjected to fragmentation and labeling using the GeneChip™ WT PLUS Reagent Kit (Thermo Fisher Scientific, Waltham, MA, USA) and then hybridized onto an Affymetrix Human Gene 2.1 ST Array Strip. Hybridization and subsequent fluidics and scanning steps were performed with an Affymetrix GeneAtlas™ System. The preliminary analysis and quality control of the scanned chips was performed using Affymetrix GeneAtlas Operating Software. The obtained CEL files were imported into BioConductor software, which is based on the statistical R programming language. For background correction, normalization, and summation of raw data, the Robust Multiarray Averaging (RMA) algorithm implemented in the “affy” package of BioConductor was applied. Biological annotation was obtained from the BioConductor “oligo” package in which the annotated data frame object was merged with the normalized data set, leading to a complete gene data table. The selection criteria for significantly changed gene expression were based on the expression fold difference higher than |2|.

Functional annotation clustering of differentially expressed genes was performed using DAVID Direct (Database for Annotation, Visualization, and Integrated Discovery) 25. Gene symbols for up- and downregulated genes from each of the compared groups were loaded into DAVID using the “RDAVIDWebService” BioConductor package. Functional annotation chats generated by DAVID with overrepresented gene annotations are shown as bubble plots from the BACA BioConductor package (https://cran.r-project.org/web/packages/BACA/BACA.pdf). Bubble plots were generated with the following criteria: *p* value < 0.1, adjusted method = Benjamini, and minimal number of genes per group = 5. Groups of genes fulfilling the mentioned criteria are presented in a graph in which the bubble size indicates the number of genes represented in the corresponding annotation and the condition of these genes in terms of their down- and upregulation.

### Histological staining

Fibroblasts were cultured on the cover slips, fixed in methanol. For the morphological analysis of extracellular matrix components, the cell cultures were stained with Azan trichrome (Bio-Optica Milano, Italy) and Sirius Red (Direct Red 80 Sigma Aldrich - 0.1% of Sirius Red in saturated aqueous picric acid), as previously described by Junqueira et al. [1979] 26 to visualize collagen fibers. When binding to collagen fibers, Sirius red molecules increase their birefringence. In polarized light, the thickest collagen fibers appear yellow/orange while the thinnest (including reticular fibers) are green. Moreover, the picric acid (a hydrophobic anionic stain) facilitates the staining by colouring in yellow. The elastic fibers were identified using Weigert's method (Weigert's for elastic fibers, Bio-Optica Milano, Italy), and Safranin O/Fast green (Sigma-Aldrich Sp. z.o.o., Poznan, Poland). All histochemical reactions were carried out according to protocols recommended by the manufacturers.

To present the components of dermis *ex vivo*, histological slides were stained with hematoxylin and eosin (H-E). Like the cell cultures, the slides were stained with Sirius Red and silver impregnation was performed to visualize reticular fibers (Bio-Optica Milano, Italy).

Immunohistochemistry (IHC) was performed to identify fibrillin-1 in the dermis. The mouse anti-human fibrillin-1 (AbD Serotec, Biogenesis, UK) monoclonal antibody (1:50) was used. Additionally, P450arom: MCA 2077T (Serotec Ltd., Kidlington, Oxford, UK) at a final dilution of 1:100 was used to identify immunolocalization cytochrome P450 aromatase. The deparaffinized sections of skin were microwaved in citrate buffer (pH 6.0) for heat-induced epitope retrieval. After slow cooling to room temperature, the slides were washed twice in PBS for 5 min and then incubated for 60 min with primary mouse anti-human fibrillin-1 antibody (AbD Serotec MorphoSys AbD GmbH, Germany). Next, the sections were stained with an avidin-biotin-peroxidase system using diaminobenzidine as the chromogen (Dako LSAB+ System-HRP: Code K0679 DakoCytomation, Denmark) in accordance with the staining procedure instruction suggested by the manufacturer. The sections were washed in distilled H_2_O and counterstained with hematoxylin. Negative control specimens were processed in the absence of primary antibody. Positive staining was defined microscopically by the visual identification of brown pigmentation.

All histochemical and IHC reactions were carried out according to protocols recommended by the manufacturers.

## Results

### Cell culture

**Figure 1:**
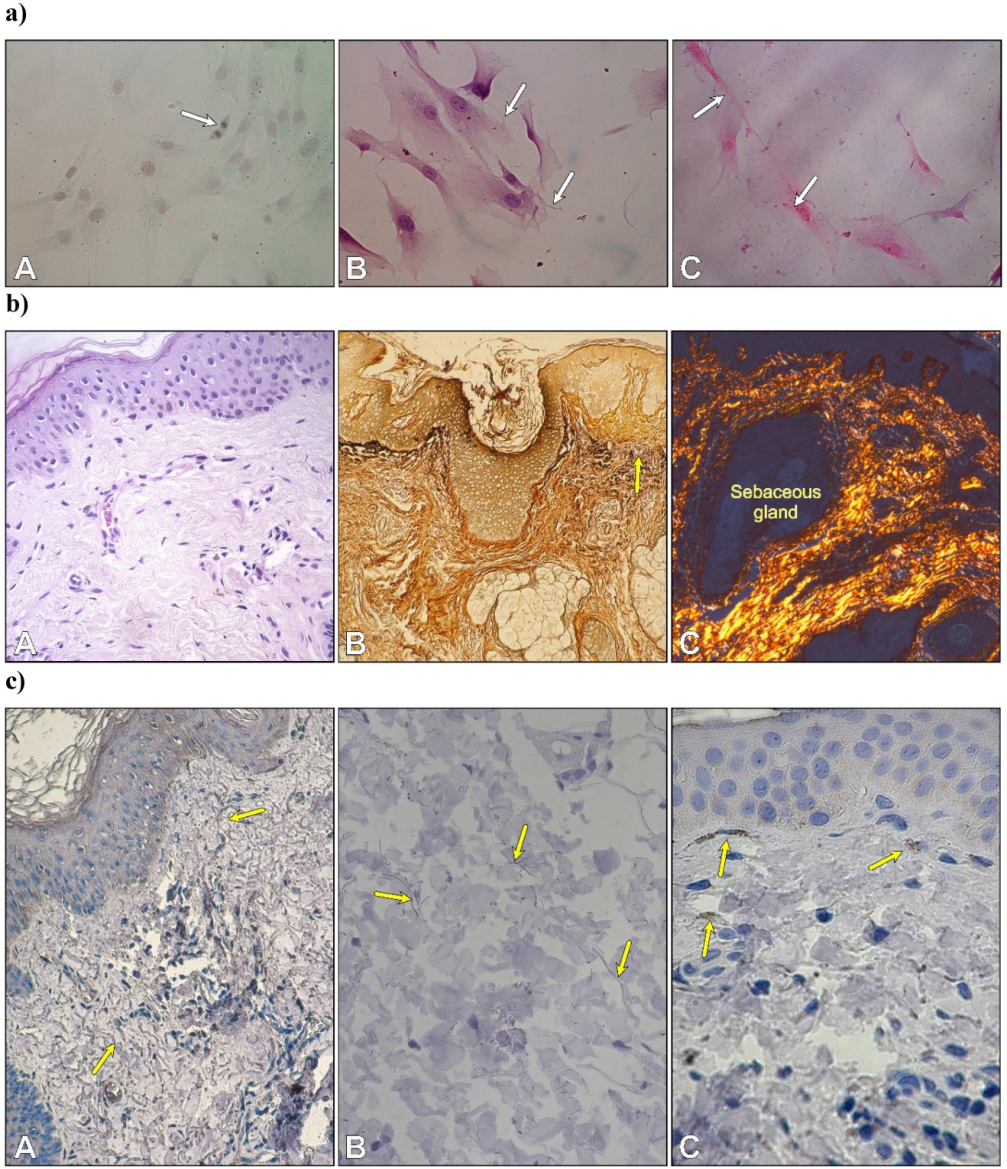
** a)** Autologous fibroblasts in the second passage. Cell division in a monolayer of fibroblasts (A); thin elastic fibers between cells (B); and collagen type I with a red-yellowish color and strong birefringence (C). Safranin O/Fast green; B. Weigert's method; C. Sirius Red. Objective magnification: x 40. **b)** Morphology of skin. Arrangement of type I collagen bundles in the papillary dermis and the reticular dermis (A) stained with H-E; localization of type III collagen (reticular fibers) after silver staining (B) and thin collagen bundles in the dermis with predominantly yellowish color (C). (A x 20; B x10, C x 10). **c)** Immunolocalization of fibrillin-1-rich microfibrils (yellow arrows) (A, B) and cytochrome P450 aromatase in fibroblasts -yellow arrows (C). (A x 20; B x 40. C x 40).

After three days of primary culture, fibroblasts adhered to glass coverslips to form a monolayer. Numerous mitotic figures were visible in fibroblasts of second passage cells stained with Safranin O/Fast green (Fig. 1aA). Histochemical staining revealed both types of fibrillary proteins in the extracellular matrix produced by fibroblasts. Very thin elastic fibers were visible between cultured cells (Fig. 1aB). Cell cultures stained with picrosirius red were analyzed by polarized microscopy. The staining displayed the presence of collagen type 1 within the cytoplasm in the form of small granules with red-yellowish staining and strong birefringence and in the extracellular space in an elongated form that was also red-yellowish in color with strong birefringence (Fig. 1aC).

### Differential Gene Expression Profiles

**Figure 2:**
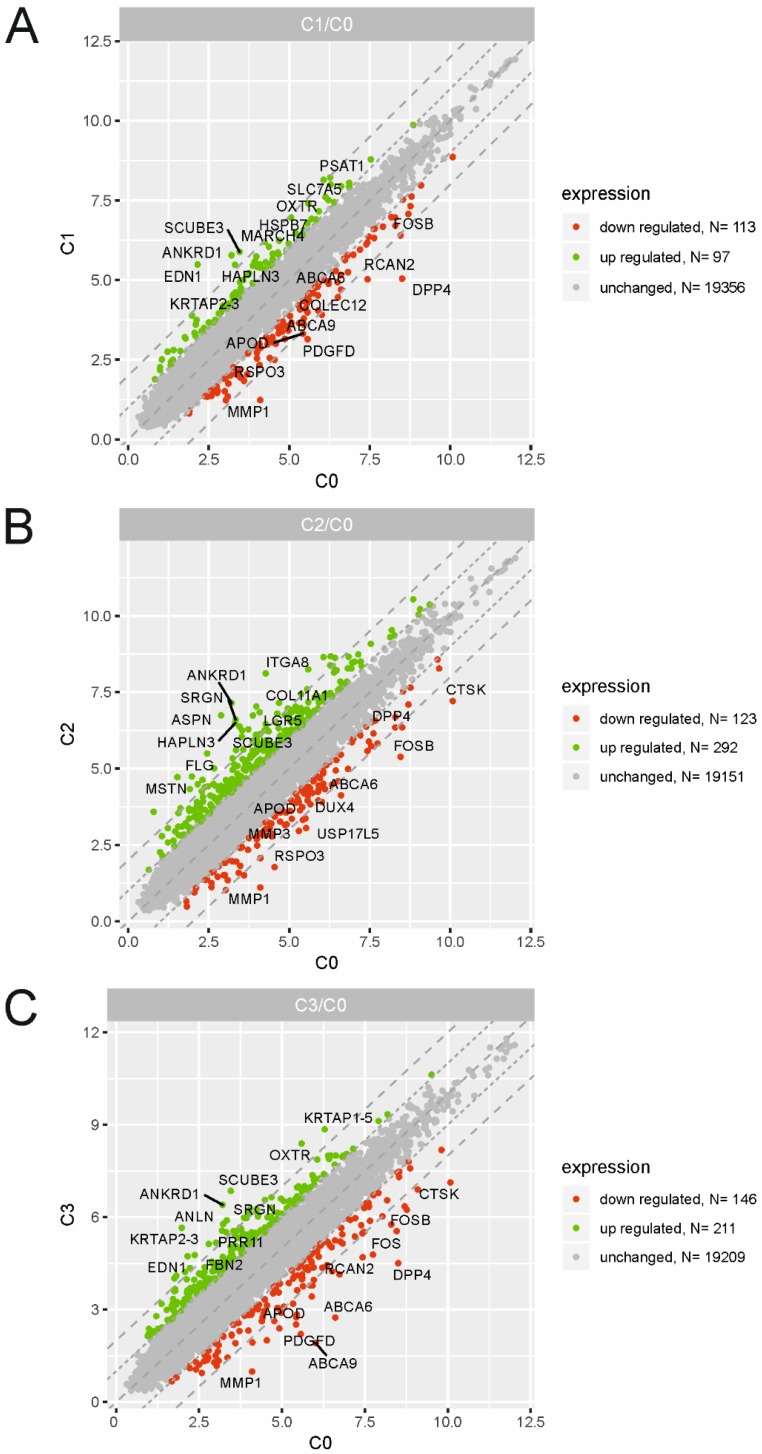
Scatter plots illustrating differentially expressed genes (DEGs) in the three experimental comparison groups: C1 vs. C0 (A), C2 vs. C0 (B), C3 vs. C0 (C). Each dot represents a single gene. The selection criteria for significantly changed gene expression were based on a greater than |2| fold difference in expression.

To identify the molecular changes encountered by cultured dermal fibroblasts from a male volunteer, we performed a detailed analysis of the differentially expressed genes (DEGs) of fibroblasts from the 1^st^ (C1), 2^nd^ (C2) and 3^rd^ (C3) cell passages compared with those in the primary culture (C0). We defined DEGs as differentially expressed with a fold change ≤ -2 (downregulated genes) and fold change ≥ 2 (upregulated genes). The results from three experimental groups (C1 vs. C0, C2 vs. C0 and C3 vs. C0) are presented as scatter plots in Fig. 2A, B, C. The highest rate of differentially expressed genes was observed in fibroblasts from the 2^nd^ passage of cells compared with the primary culture. We found that a total of 415 genes were regulated in C2 compared with the C0 group, including 292 upregulated genes and 123 downregulated genes. Under identical experimental conditions, microarray analysis revealed 357 DEGs in the C3 vs. C0 groups (211 up-, 146 downregulated) and only 210 overrepresented genes in C1 compared with the C0 group (97 up-, 113 downregulated). The top 10 genes with the highest and lowest fold change values from each of the three experimental comparisons are listed in Table 1. The complete list of up- and downregulated genes is included in the supplementary material (Table S1, S2 and S3).

**Figure 3:**
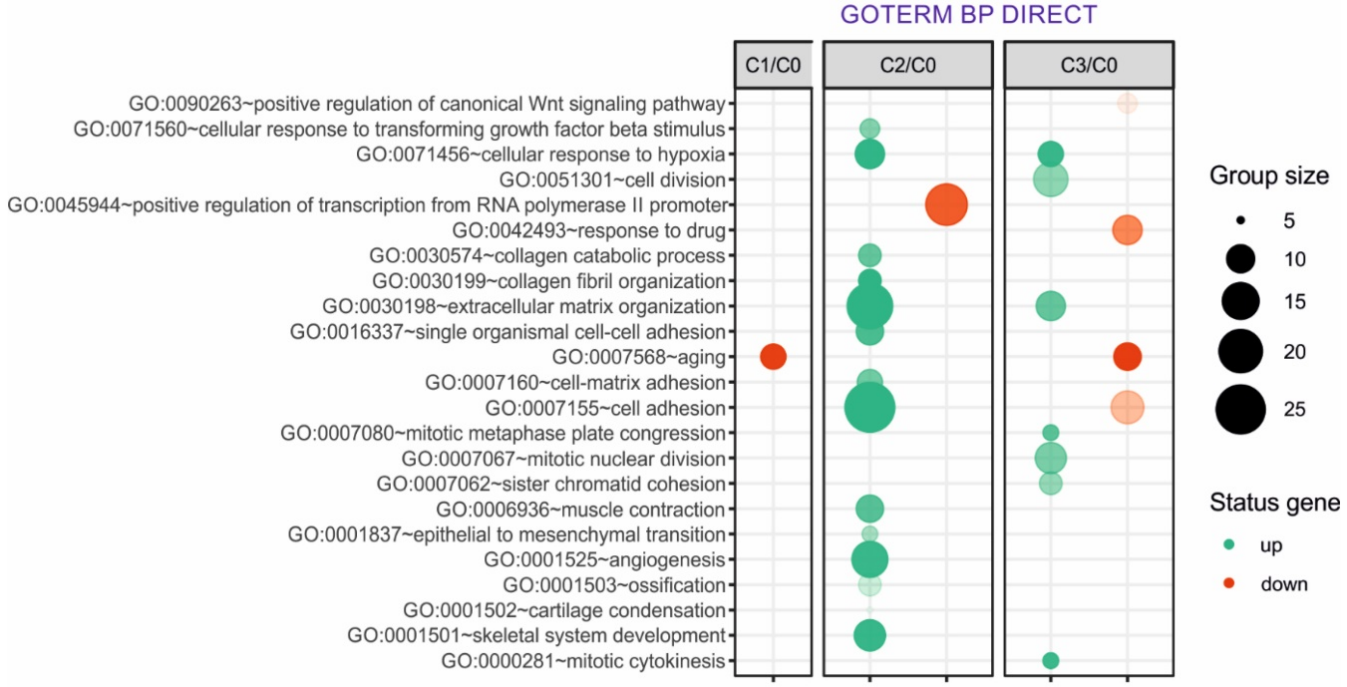
Overrepresented biological processes assigned according to Gene Ontology (GO) classification in cultured skin fibroblasts from the 1^st^ (C1), 2^nd^ (C2) and 3^rd^ (C3) passages compared with the primary culture (C0). Groups of genes fulfilling the criteria: adjusted *p-*value < 0.1, method = Benjamini, and minimum number of genes per group = 5, are presented in a graph in which the bubble size indicates the number of genes represented in the corresponding annotation and the condition of these genes in terms of their up- and downregulation. The transparency of the bubbles reflects the *p-*value (more transparent is closer to the border of p = 0.1).

**Figure 4:**
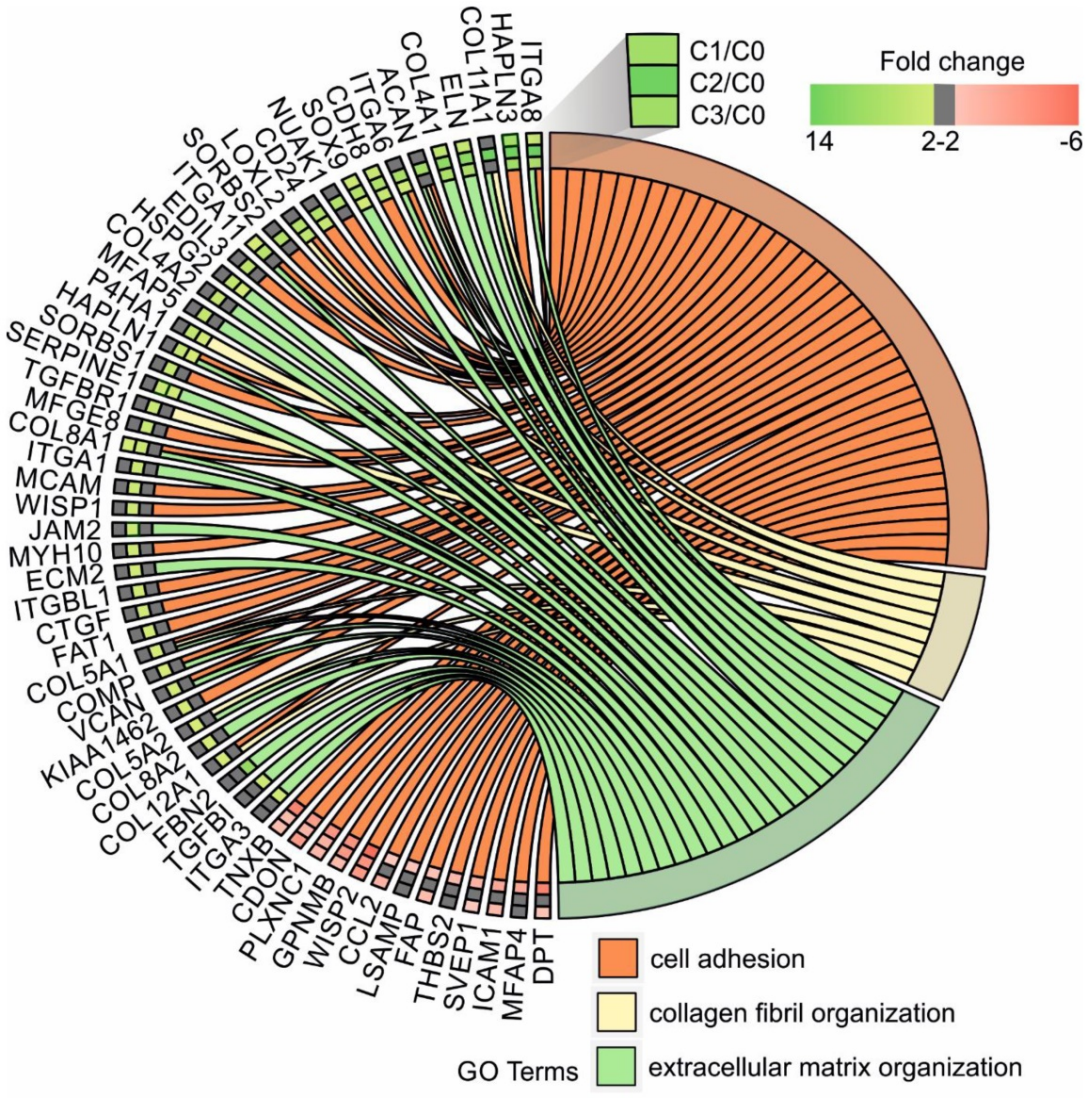
Circular genome data visualization (circos) plot for the selected overrepresented GO terms and corresponding differentially expressed genes (DEGs). The relevant fold change values are presented by the color scale (green - upregulated, red - downregulated, grey - unchanged), where external rectangles indicate C1 vs. C0, inner rectangles indicate C2 vs. C0 and internal rectangles indicate C3 vs. C0 comparison groups.

Next, up- and downregulated genes from all experimental groups were assigned Gene Ontology terms for biological processes (GO term BP) classification. The selection criteria for significantly changed groups of genes were as follows: p-value < 0.1, adjusted method = Benjamini, and minimal number of genes per group = 5. From the complete list of overrepresented terms displayed as in bubble diagram presented in Fig. 3, we selected three biological processes relevant to fibroblast function in the context of autologous cell skin transplantation. The results for the biological terms “GO:0007155 - cell adhesion”, “GO: 0030199 - collagen fibril organization” and “GO: 0030198 - extracellular matrix organization” are presented as a circos (circular genome data visualization) plot (Fig. 4).

In our study, the majority of upregulated genes were identified in the C2 group. These genes included the “cell adhesion” category and the most diversified group of genes based on the fold change value differences (Fig. 3). The genes associated with “cell adhesion” are shown in circular genome data visualization (circos) plot (Fig. 4) (n = 39 genes fulfilling the selection criteria), and their division based on the regulation form is presented in Table 2. These genes are mainly responsible for the attachment of cells via cell - cell adhesion (*KIAA1462*, *EDIL3*, *MFGE8*, *MCAM*, *MYH10*, *ITGBL1*, *FAT1*) or cell - extracellular matrix interactions (*ACAN*, *VCAN*, *SORBS1*, *SORBS2*, *WISP1*, *CTGF*, *COMP* ). On the other hand, some of the genes that mapped to the “cell adhesion” term were downregulated in at least two experimental groups, e.g., *TNXB*, encoding a member of the tenascin family of glycoproteins, which has anti-adhesive properties, and the *FAP* gene, for which the protein product is a serine protease involved in extracellular matrix degradation (Fig. 4, Table 2).

The process with the second highest accumulation of upregulated genes was “extracellular matrix organization”, which was observed in the 2^nd^ passage of fibroblasts (Fig. 3). “Extracellular matrix organization” comprises genes that are responsible for the assembly and rearrangement of the three-dimensional network of extracellular macromolecules. Most of these genes were significantly upregulated in cultured human skin fibroblasts from the 2^nd^ passage (C2) compared with those from the primary culture (C0) (Table 2), whereas the fold change value of those genes remained unaffected in the other experimental groups. This finding applies to genes encoding proteoglycans (*ACAN*, *VCAN, ECM2),* collagens (*COL4A2*, *COL5A1, COL5A2, COL8A2*), microfibril (*MFAP5*), integrin (*ITGA1*), adhesion molecule (*JAM2*), and thrombospondin (*COMP* ). An exception to this observation is the expression of the *FBN2* (fibrillin 2), *TGFBI* (transforming growth factor, beta-induced) and *ITGA3* (integrin, alpha 3) genes, which only exceeded the accepted threshold in fibroblasts from the 3^rd^ cell passage (C3 vs. C0) (Fig. 4).

The expression of genes that had the “collagen fibril organization” annotation showed an interesting pattern. Starting from the C2 cell passage, we observed induction of genes involved in the synthesis of collagen fibrils, such as *COL5A1*, *COL8A2*, *COL11A1*, *COL12A1*, *ACAN* and *TGFBR1* (Fig. 4, Table 2). In addition, genes determining the trimerization of collagen chains and formation of elastic fibers (*P4HA1*, *LOXL2*) were highly upregulated in skin fibroblasts from both the 2^nd^ and 3^rd^ passages compared with those from the primary culture. The expression of the aforementioned genes in C1 compared with that in the C0 group remained unchanged (Fig. 4).

Noticeably, due to the ambiguous nature of the Gene Ontology database, some of the genes are assigned to more than one functional annotation, i.e., *ITGA6*, *ITGA8*, *ITGA11*, *HAPLN1*, *COL8A1*, *COMP* and *VCAN* genes, which are mapped to “GO:0007155 - cell adhesion” and “GO:0030198 - extracellular matrix organization” (Fig. 4, Table 2).

## Discussion

The structural integrity and function of skin depends on elements of extracellular matrix organization that are produced and released by dermal fibroblasts. Similar to other organs, human skin undergoes natural aging processes, in which major alternations are localized in the dermal extracellular matrix. Therefore, the appearance of skin is the first sign of aging 4. Recently, increased interest in the prevention of skin aging and the maintenance and improvement of its quality has gained particular attention 24, 27.

Volume loss due to facial aging can be restored by facial volumization using a variety of materials as soft tissue fillers, including biodegradable products (hyaluronic acid, collagen, autologous fat, etc.) 28, 29. The use of autologous tissues is becoming increasingly popular since tissues represent the most convenient materials as they do not trigger biological reactions 28. An innovative method to correct age-related skin changes is the use of cultured fibroblasts derived from the patient's own skin 24. In our previous study, 24 we transplanted a suspension of autologous fibroblasts collected from the 4^th^ passage of an *ex vivo* culture into the postauricular area of skin in male volunteers. A remarkable improvement in dermal morphology was noted three months after administration of the fibroblasts. We observed a substantial increase in the number of fibroblasts, a significant increase of the diameter of the collagen fiber bundles, and an improvement in the density of reticular fibers, fibrillin-1 rich microfibrils, and elastic fibers 24.

In the present study, we aimed to establish the best population of autologous fibroblasts in terms of their proliferative and secretory properties and to investigate the transcriptome profile of cells from three consecutive passages compared with those from the primary culture. The morphological evaluation of fibroblasts from the primary culture and the subsequent 1^st^, 2^nd^ and 3^rd^ passages indicated that fibroblasts of the second passage met the most expectations regarding their proliferation and secretion capabilities. Morphology of skin of patient in the study was the similar to that in previous untreated patient in the same age 24. Fang et al. indicated that fibroblasts isolated from human foreskin preserve typical morphologic characteristics with proliferative and secretory activities without genetic abnormalities for five consecutive passages 30. Similar results were obtained by Zeng et al. for cultured fibroblasts isolated from human skin from both sexes undergoing blepharoplasty 31. The cells maintained proliferative and secretory activity during culture in subsequent passages, and cells before the 5^th^ passage were the most applicable for clinical use. Furthermore, the genomic stability of the cultured cells was also maintained between passages 5 and 10 31. This finding is in accordance with a historical study by Hayflik and Moorhead, who showed that human fibroblasts could maintain genomic stability even after 40 generations 32. In light of the results of the above studies, we expect that genomic stability was also maintained by the fibroblasts used in our current study.

To select the best fibroblast population for clinical purposes, we performed a detailed analysis of the differentially expressed genes (DEGs) of the cultured cells after the 1^st^, 2^nd^, and 3^rd^ passage compared with genes from cells of the primary culture. We found the highest number of upregulated genes in the cells at the 2^nd^ passage compared with those at 1^st^ and 3^rd^ passages (292, 97, and 211, respectively). Genes directly related to dermal ECM organization, such as ASPN (asporin), SRGN (serglycin), and HAPLN3 (hyaluronan and proteoglycan link protein 3), were among the top 10 genes with the highest fold expression change in fibroblasts from the 2^nd^ passage.

Asporin belongs to the leucine-rich repeat (LRR) superfamily of proteins 33. Our study revealed that asporin was only upregulated in cells after the second passage (14.50-fold change). Cloning of human and mouse asporin cDNAs revealed that the protein is closely related to decorin and biglycan 34. Asporin is a secretory product of fibroblasts in the human dermis, and its role is similar to that of decorin. By binding to type I collagen fibrils, the protein ensures appropriate assembly of the fibrils and inhibits the cleavage of collagen fibrils by matrix metaloproteinase-1 35.

Serglycin (9.67-fold change; 5.93 in C3) is a widely distributed proteoglycan that was previously assumed to be hematopoietic cell specific and is known as a hematopoietic proteoglycan core protein 36. Serglycin mRNA is expressed outside the hematopoietic cell system. High levels of serglycin mRNA were detected in endothelial cells and smooth muscle cells, whereas low levels were detected in fibroblasts isolated from human skin 37, 38. BB Werth (2011) demonstrated that single-dose UVB irradiation in combination with IL-1alpha induced serglycin mRNA in cultured human dermal fibroblasts 38. Thus, serglycin is primarily important in inflammatory, allergic, and immune reactions in skin 39 and the cutaneous response to UV irradiation 38.

The third HAPLN3 gene upregulated (8.98-fold change; 4.47 in C1) in fibroblasts at the second passage encodes hyaluronan and proteoglycan link protein 3 and belongs to the hyaluronan and proteoglycan link protein family. This gene family plays an important role in the construction and stabilization of the hyaluronan-dependent extracellular matrix 40, 41. Gene Ontology (GO) annotations related to this gene include extracellular matrix structural constituent and hyaluronic acid binding [https://www.genecards.org/cgi-bin/carddisp.pl?gene=HAPLN].

Furthermore, we found two genes encoding he collagen-metabolizing enzymes MMP3 (-5.07-fold change) and MMP1 (-7.96-fold change; C1 -7.29; C3 -8.62) among the 10 genes with the lowest fold change in expression in fibroblasts from the 2^nd^ passage. Proteins of the matrix metalloproteinase (MMP) family produced by several different types of cells in skin, including fibroblasts, are involved in the breakdown of the extracellular matrix in normal physiological processes. Controlled breakdown of the ECM by MMPs plays an important role in the detachment and migration of cells as well as tissue remodeling in several physiological processes 42. The human MMP3 gene encodes stromelysin-1, which is also known as matrix metalloproteinase-3 (MMP-3). MMP-3 is an enzyme that degrades collagen types II, III, IV, IX, and X; proteoglycans; gelatin; fibronectin; laminin; and fibrillin-1 43, 44. The other upregulated gene, MMP1, encodes matrix metalloproteinase-1 (MMP-1), which is also known as interstitial collagenase and fibroblast collagenase. MMP-1 is the major collagenase that degrades native fibrillary collagen types I, II, III, V, and XI in extracellular spaces 43. The degradation of type I collagen is initiated by interstitial collagenase, creates space for cells to migrate, and is necessary for epithelial cell migration and wound healing in culture models 45-47.

The bubble diagram indicates that the secretory properties of fibroblasts from the 2^nd^ passage are the best given the highest accumulation of upregulated genes that regulate critical processes for skin homeostasis. These genes are strongly associated with the processes that regulate aging and reflect the best properties of fibroblasts regarding their potential clinical application in esthetic medicine. According to the Gene Ontology (GO) classification in the cultured skin fibroblasts from the 2^nd^ (C2) passage, the most numerous upregulated genes exhibiting the best properties for autologous transplantation include genes responsible for the following processes: (i) GO: 0007155 - cell adhesion (group size 25), (ii) GO: 0030198 - extracellular matrix organization (group size 20), and (iii) GO: 0030199 - collagen fibril organization (group size 10). Of note, since the third group of upregulated genes is involved in extracellular matrix organization, we additionally focused on that group, although fewer upregulated genes were observed in this group compared with other groups.

Within the cell adhesion category, 39 genes were taken into consideration and are presented in a circular genome data visualization plot (Fig. 4). The majority of upregulated genes that were the most suitable for clinical use were identified within the C2 group. These genes are mainly responsible for the attachment of cells via cell-cell adhesion (*KIAA1462*, *EDIL3*, *MFGE8*, *MCAM*, *MYH10*, *ITGBL1*, *FAT1, HAPLN3, ITGA8*) or cell-extracellular matrix interactions (*ACAN*, *VCAN*, *SORBS1*, *SORBS2*, *WISP1*, *CTGF*, *COMP, ITGA11*). *KIAA1462* is crucial in cell-cell junctions 48. The aforementioned genes exhibit a large spectrum of properties and regulate important processes. An increasing number of studies have been performed, and new roles of separate genes are known.

In addition, less than 15% of genes associated with the cell adhesion category are downregulated, e.g., *TNXB* encodes tenascins, which exhibit anti-adhesive effects and are also downregulated. Tenascin X (TNX) is a glycoprotein that regulates tissue structure through anti-adhesive interactions with collagen in the extracellular matrix 49.

Regarding genes involved in extracellular matrix organization, circular genome data visualization plots demonstrate that the most numerous genes, including *COL8A1, COL8A2, COL5A1, COL5A2, COL4A1,* and *COL4A2*, are upregulated in the fibroblasts from the 2^nd^ passage. These genes are involved in collagen synthesis. It is commonly known that collagen is one of the most abundant and most important proteins in the human dermal extracellular matrix 50.

Genes responsible or involved in collagen fibril organization processes are upregulated within cells from the 2^nd^ passage compared with C0 cells. These genes include *COL12A1, COL5A2, COL5A1, TGFBR1, P4HA1, LOXL2, ACAN,* and* COL11A1.* Collagen fibrils are unique components in most human tissues, including skin 51, and their organization is extremely important, especially regarding the potential clinical application of autologous fibroblasts.

## Conclusion

In this study, transcriptome profile analysis indicated that the second passage seems to be optimal for the preparation of fibroblasts for potential autologous transplantation to the skin essentially due to the secretory activity of the cells and extracellular matrix organization. Moreover, the same cell population reveals repression of genes encoding proteinases responsible for the degradation of dermal extracellular matrix proteins. Altogether, this study provides evidence for the promising mRNA profile of human fibroblasts for treating various forms of skin aging. However, further and more extensive study could be noteworthy focusing on the processes of differentiation, cell cycle and cell proliferation status.

## Supplementary Material

Supplementary tables.

## Figures and Tables

**Table 1:** Top 10 genes with the highest and lowest values of the fold expression change within the three experimental comparison groups: C1 vs. C0, C2 vs. C0, C3 vs. C0.

Comparison group	Gene symbol	Gene name	Entrez Gene ID	Fold change
**C1 vs.C0**	*EDN1*	endothelin 1	1906	10.09
	*ANKRD1*	ankyrin repeat domain 1 (cardiac muscle)	27063	5.95
	*SCUBE3*	signal peptide. CUB domain. EGF-like 3	222663	5.38
	*HAPLN3*	hyaluronan and proteoglycan link protein 3	145864	4.47
	*SLC7A5*	solute carrier family 7 (amino acid transporter light chain. L system). member 5	8140	4.23
	*MARCH4*	membrane-associated ring finger (C3HC4) 4. E3 ubiquitin protein ligase.	57574	3.91
	*PSAT1*	phosphoserine aminotransferase 1	29968	3.86
	*KRTAP2-3*	keratin associated protein 2-3	730755	3.76
	*HSPB7*	heat shock 27kDa protein family. member 7 (cardiovascular)	27129	3.74
	*OXTR*	oxytocin receptor	5021	3.52
	*ABCA6*	ATP-binding cassette. sub-family A (ABC1). member 6	23460	-3.74
	*RSPO3*	R-spondin 3	84870	-4.14
	*COLEC12*	collectin sub-family member 12	81035	-4.14
	*FOSB*	FBJ murine osteosarcoma viral oncogene homolog B	2354	-4.16
	*ABCA9*	ATP-binding cassette. sub-family A (ABC1). member 9	10350	-4.29
	*APOD*	apolipoprotein D	347	-4.30
	*RCAN2*	regulator of calcineurin 2	1023 1	-5.30
	*PDGFD*	platelet derived growth factor D	80310	-5.37
	*MMP1*	matrix metallopeptidase 1 (interstitial collagenase)	43 12	-7.29
	*DPP4*	dipeptidyl-peptidase 4	1803	-10.99
**C2 vs.C0**	*ANKRD1*	ankyrin repeat domain 1 (cardiac muscle)	27063	15.34
	*ASPN*	asporin	54829	14.50
	*ITGA8*	integrin. alpha 8	8516	14.40
	*SRGN*	serglycin	5552	9.67
	*MSTN*	myostatin	2660	9.15
	*HAPLN3*	hyaluronan and proteoglycan link protein 3	145864	8.98
	*COL11A1*	collagen. type XI. alpha 1	1301	8.34
	*FLG*	filaggrin	2312	8.23
	*SCUBE3*	signal peptide. CUB domain. EGF-like 3	222663	7.56
	*LGR5*	leucine-rich repeat containing G protein-coupled receptor 5	8549	7.30
	*APOD*	apolipoprotein D	347	-4.30
	*DUX4*	double homeobox 4	100288687	-4.39
	*DPP4*	dipeptidyl-peptidase 4	1803	-4.45
	*MMP3*	matrix metallopeptidase 3 (stromelysin 1. progelatinase)	4314	-5.07
	*USP17L5*	ubiquitin specific peptidase 17-like family member 5	728386	-5.56
	*ABCA6*	ATP-binding cassette. sub-family A (ABC1). member 6	23460	-5.60
	*RSPO3*	R-spondin 3	84870	-6.83
	*CTSK*	cathepsin K	1513	-7.29
	*MMP1*	matrix metallopeptidase 1 (interstitial collagenase)	43 12	-7.96
	*FOSB*	FBJ murine osteosarcoma viral oncogene homolog B	2354	-8.42
**C3 vs. C0**	*KRTAP2-3*	keratin associated protein 2-3	730755	12.81
	*SCUBE3*	signal peptide. CUB domain. EGF-like 3	222663	10.53
	*ANKRD1*	ankyrin repeat domain 1 (cardiac muscle)	27063	9.15
	*OXTR*	oxytocin receptor	5021	6.97
	*EDN1*	endothelin 1	1906	5.99
	*SRGN*	serglycin	5552	5.93
	*KRTAP1-5*	keratin associated protein 1-5	83895	5.92
	*ANLN*	anillin. actin binding protein	54443	5.85
	*FBN2*	fibrillin 2	2201	5.27
	*PRR11*	proline rich 11	55771	5.18
	*RCAN2*	regulator of calcineurin 2	10231	-6.64
	*APOD*	apolipoprotein D	347	-7.50
	*FOSB*	FBJ murine osteosarcoma viral oncogene homolog B	2354	-7.52
	*CTSK*	cathepsin K	1513	-7.73
	*FOS*	FBJ murine osteosarcoma viral oncogene homolog	2353	-7.77
	*MMP1*	matrix metallopeptidase 1 (interstitial collagenase)	4312	-8.62
	*PDGFD*	platelet derived growth factor D	80310	-10.27
	*ABCA6*	ATP-binding cassette. sub-family A (ABC1). member 6	23460	-14.56
	*DPP4*	dipeptidyl-peptidase 4	1803	-15.95
	*ABCA9*	ATP-binding cassette. sub-family A (ABC1). member 9	10350	-17.06

**Table 2:**
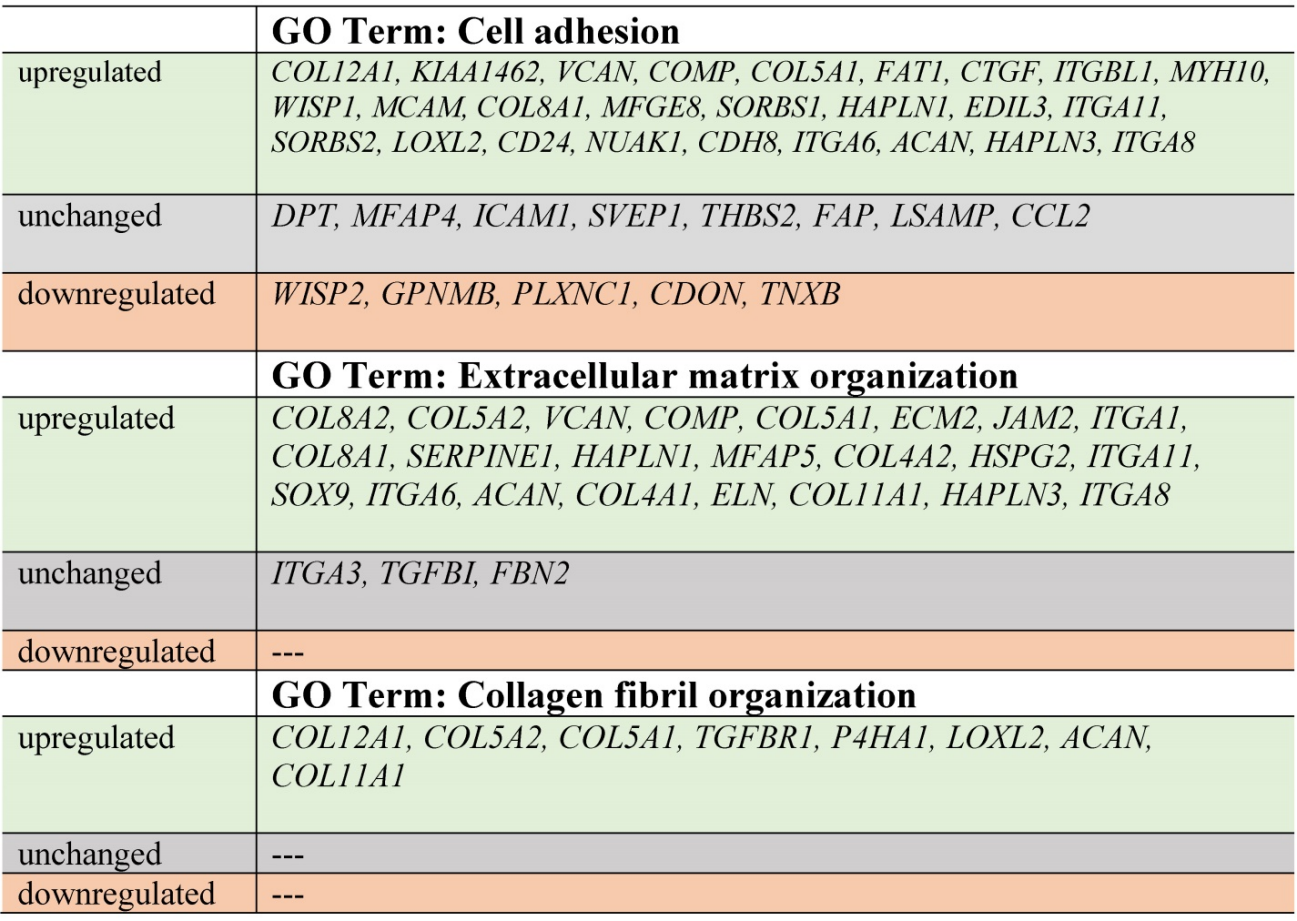
Distribution of genes in fibroblasts from the 2^nd^ passage according to the selected processes.
