# Supplementary material for: Transcriptome Profile of Human Fibroblasts in an Ex Vivo Culture
Source: Int J Med Sci. 2020 Jan 1;17(1):125–36. doi: 10.7150/ijms.35693 (PMC6945561; doi:10.7150/ijms.35693)
Supplement: Supplementary file 1 — Supplementary tables. [file ijmsv17p0125s1.pdf]

Table S1. Complete list of up- and down-regulated genes in C1 cells.

Direction in C1 cells	Gene symbol	Gene name	Entrez Gene ID	Fold change C1 vs C0
up	EDN1	endothelin 1	1906	10,09
up	ANKRD1	ankyrin repeat domain 1 (cardiac muscle)	27063	5,95
up	SCUBE3	signal peptide, CUB domain, EGF-like 3	222663	5,38
up	HAPLN3	hyaluronan and proteoglycan link protein 3	145864	4,47
up	SLC7A5	solute carrier family 7 (amino acid transporter light chain, L system), member 5	8140	4,23
up	MARCH4	membrane-associated ring finger (C3HC4) 4, E3 ubiquitin protein ligase	57574	3,91
up	PSAT1	phosphoserine aminotransferase 1	29968	3,86
up	KRTAP2-3	keratin associated protein 2-3	730755	3,76
up	HSPB7	heat shock 27kDa protein family, member 7 (cardiovascular)	27129	3,74
up	OXTR	oxytocin receptor	5021	3,52
up	ASNS	asparagine synthetase (glutamine-hydrolyzing)	440	3,39
up	KRTAP1-5	keratin associated protein 1-5	83895	3,27
up	CHAC1	ChaC, cation transport regulator homolog 1 (E, coli)	79094	3,22
up	DCLK2	doublecortin-like kinase 2	166614	3,15
up	SLC16A4	solute carrier family 16, member 4	9122	3,08
up	LGR5	leucine-rich repeat containing G protein-coupled receptor 5	8549	2,99
up	COL4A1	collagen, type IV, alpha 1	1282	2,94
up	POTEI	POTE ankyrin domain family, member I	653269	2,89
up	GSTT2	glutathione S-transferase theta 2	2953	2,84
up	ULBP1	UL16 binding protein 1	80329	2,81
up	TPM1	tropomyosin 1 (alpha)	7168	2,78
up	CHRM2	cholinergic receptor, muscarinic 2	1129	2,77
up	PDLIM5	PDZ and LIM domain 5	10611	2,76
up	PLN	phospholamban	5350	2,75
up	ADIRF	adipogenesis regulatory factor	10974	2,72
up	ELN	elastin	2006	2,71
up	SYNPO2	synaptopodin 2	171024	2,68
up	B3GALT2	UDP-Gal:betaGlcNAc beta 1,3-galactosyltransferase, polypeptide 2	8707	2,67
up	CDH8	cadherin 8, type 2	1006	2,66
up	FCGR1B	Fc fragment of IgG, high affinity Ib, receptor (CD64)	2210	2,64
up	MALL	mal, T-cell differentiation protein-like	7851	2,62
up	CALD1	caldesmon 1	800	2,52
up	TM4SF1	transmembrane 4 L six family member 1	4071	2,52
up	GPC4	glypican 4	2239	2,46
up	STC2	stanniocalcin 2	8614	2,45
up	ITGA8	integrin, alpha 8	8516	2,44

up	LGMN	legumain	5641	2,43
up	SEL1L3	sel-1 suppressor of lin-12-like 3 (C, elegans)	23231	2,43
up	SOX9	SRY (sex determining region Y)-box 9	6662	2,43
up	MIR3191	microRNA 3191	100422832	2,43
up	SRGN	serglycin	5552	2,42
up	CXorf56	chromosome X open reading frame 56	63932	2,42
up	GLS	glutaminase	2744	2,39
up	LRRC37A2	leucine rich repeat containing 37, member A2	474170	2,38
up	TSPAN18	tetraspanin 18	90139	2,38
up	RASA4	RAS p21 protein activator 4	10156	2,38
up	FAM101B	family with sequence similarity 101, member B	359845	2,36
up	PPP1R3C	protein phosphatase 1, regulatory subunit 3C	5507	2,34
up	GREM2	gremlin 2, DAN family BMP antagonist	64388	2,33
up	PHGDH	phosphoglycerate dehydrogenase	26227	2,33
up	GATA6	GATA binding protein 6	2627	2,31
up	OR8H3	olfactory receptor, family 8, subfamily H, member 3	390152	2,28
up	SLC7A11	solute carrier family 7 (anionic amino acid transporter light chain, xc- system), member 11	23657	2,28
up	VASP	vasodilator-stimulated phosphoprotein	7408	2,25
up	LYPD6B	LY6/PLAUR domain containing 6B	130576	2,25
up	AFF3	AF4/FMR2 family, member 3	3899	2,24
up	PDLIM7	PDZ and LIM domain 7 (enigma)	9260	2,24
up	C5orf28	chromosome 5 open reading frame 28	64417	2,23
up	LUZP2	leucine zipper protein 2	338645	2,22
up	C19orf25	chromosome 19 open reading frame 25	148223	2,20
up	PSMD3	proteasome (prosome, macropain) 26S subunit, non-ATPase, 3	5709	2,20
up	DSP	desmoplakin	1832	2,19
up	C11orf87	chromosome 11 open reading frame 87	399947	2,19
up	SASS6	spindle assembly 6 homolog (C, elegans)	163786	2,18
up	CDH2	cadherin 2, type 1, N-cadherin (neuronal)	1000	2,17
up	BMP6	bone morphogenetic protein 6	654	2,17
up	PCK2	phosphoenolpyruvate carboxykinase 2 (mitochondrial)	5106	2,17
up	ASPN	asporin	54829	2,16
up	LTB4R2	leukotriene B4 receptor 2	56413	2,16
up	PPP2R2B	protein phosphatase 2, regulatory subunit B, beta	5521	2,14
up	FAM43A	family with sequence similarity 43, member A	131583	2,13
up	TES	testis derived transcript (3 LIM domains)	26136	2,10
up	NOTCH3	notch 3	4854	2,10
up	LMO7	LIM domain 7	4008	2,10
up	ALDH1B1	aldehyde dehydrogenase 1 family, member B1	219	2,10
up	SPOCD1	SPOC domain containing 1	90853	2,08

up	SLC16A3	solute carrier family 16 (monocarboxylate transporter), member 3	9123	2,07
up	IGKV@	immunoglobulin kappa variable cluster	3519	2,07
up	POTEG	POTE ankyrin domain family, member G	404785	2,07
up	SPDL1	spindle apparatus coiled-coil protein 1	54908	2,07
up	WFDC1	WAP four-disulfide core domain 1	58189	2,06
up	TWISTNB	TWIST neighbor	221830	2,06
up	CNKSR2	connector enhancer of kinase suppressor of Ras 2	22866	2,06
up	TIPARP	TCDD-inducible poly(ADP-ribose) polymerase	25976	2,06
up	NPAS2	neuronal PAS domain protein 2	4862	2,04
up	MIR614	microRNA 614	693199	2,04
up	ZNF595	zinc finger protein 595	152687	2,04
up	ITGA11	integrin, alpha 11	22801	2,03
up	COL8A1	collagen, type VIII, alpha 1	1295	2,03
up	ACTA2	actin, alpha 2, smooth muscle, aorta	59	2,03
up	ENTPD4	ectonucleoside triphosphate diphosphohydrolase 4	9583	2,03
up	ABCC3	ATP-binding cassette, sub-family C (CFTR/MRP), member 3	8714	2,03
up	ALG14	ALG14, UDP-N-acetylglucosaminyltransferase subunit	199857	2,02
up	SLC2A3	solute carrier family 2 (facilitated glucose transporter), member 3	6515	2,02
up	OR4D10	olfactory receptor, family 4, subfamily D, member 10	390197	2,01
up	PAWR	PRKC, apoptosis, WT1, regulator	5074	2,00
up	DKK1	dickkopf WNT signaling pathway inhibitor 1	22943	2,00
down	PPAP2B	phosphatidic acid phosphatase type 2B	8613	-2,01
down	KIR3DL1	killer cell immunoglobulin-like receptor, three domains, long cytoplasmic tail, 1	3811	-2,01
down	NFKBIZ	nuclear factor of kappa light polypeptide gene enhancer in B-cells inhibitor, zeta	64332	-2,01
down	OXT	oxytocin/neurophysin I prepropeptide	5020	-2,01
down	LRRN4CL	LRRN4 C-terminal like	221091	-2,02
down	SERPING1	serpin peptidase inhibitor, clade G (C1 inhibitor), member 1	710	-2,02
down	SOD3	superoxide dismutase 3, extracellular	6649	-2,03
down	PPARGC1A	peroxisome proliferator-activated receptor gamma, coactivator 1 alpha	10891	-2,03
down	MXD3	MAX dimerization protein 3	83463	-2,03
down	BMPER	BMP binding endothelial regulator	168667	-2,04
down	DNM1	dynamin 1	1759	-2,05
down	C1S	complement component 1, s subcomponent	716	-2,05
down	SVEP1	sushi, von Willebrand factor type A, EGF and pentraxin domain containing 1	79987	-2,06
down	CLIC2	chloride intracellular channel 2	1193	-2,07
down	ZNF563	zinc finger protein 563	147837	-2,07
down	FADS1	fatty acid desaturase 1	3992	-2,07

down	ZNF608	zinc finger protein 608	57507	-2,10
down	CRABP2	cellular retinoic acid binding protein 2	1382	-2,10
down	ZNF14	zinc finger protein 14	7561	-2,11
down	CELF2	CUGBP, Elav-like family member 2	10659	-2,11
down	TMTC1	transmembrane and tetratricopeptide repeat containing 1	83857	-2,11
down	PTGFR	prostaglandin F receptor (FP)	5737	-2,11
down	UBC	ubiquitin C	7316	-2,12
down	SLC9A9	solute carrier family 9, subfamily A (NHE9, cation proton antiporter 9), member 9	285195	-2,12
down	SCPEP1	serine carboxypeptidase 1	59342	-2,13
down	TCEAL3	transcription elongation factor A (SII)-like 3	85012	-2,13
down	IGSF10	immunoglobulin superfamily, member 10	285313	-2,14
down	SLC40A1	solute carrier family 40 (iron-regulated transporter), member 1	30061	-2,14
down	DKK2	dickkopf WNT signaling pathway inhibitor 2	27123	-2,15
down	FLT3LG	fms-related tyrosine kinase 3 ligand	2323	-2,15
down	ZNF436	zinc finger protein 436	80818	-2,15
down	COL14A1	collagen, type XIV, alpha 1	7373	-2,15
down	JUN	jun proto-oncogene	3725	-2,16
down	PRSS36	protease, serine, 36	146547	-2,18
down	DPT	dermatopontin	1805	-2,18
down	SCARA3	scavenger receptor class A, member 3	51435	-2,19
down	PDE7B	phosphodiesterase 7B	27115	-2,22
down	ATAD3B	ATPase family, AAA domain containing 3B	83858	-2,23
down	INSIG1	insulin induced gene 1	3638	-2,23
down	SAMHD1	SAM domain and HD domain 1	25939	-2,24
down	IGLV3-10	immunoglobulin lambda variable 3-10	---	-2,25
down	HSPA6	heat shock 70kDa protein 6 (HSP70B)	3310	-2,25
down	FBLN7	fibulin 7	129804	-2,26
down	ENPP2	ectonucleotide pyrophosphatase/phosphodiesterase 2	5168	-2,27
down	DHCR24	24-dehydrocholesterol reductase	1718	-2,29
down	MYO1D	myosin ID	4642	-2,29
down	DCN	decorin	1634	-2,30
down	GLUD1	glutamate dehydrogenase 1	2746	-2,31
down	CTSK	cathepsin K	1513	-2,32
down	RRP7B	ribosomal RNA processing 7 homolog B (S, cerevisiae)	91695	-2,33
down	TNXB	tenascin XB	7148	-2,34
down	FAP	fibroblast activation protein, alpha	2191	-2,35
down	FBLN1	fibulin 1	2192	-2,37
down	OR56A5	olfactory receptor, family 56, subfamily A, member 5	390084	-2,37
down	GPNMB	glycoprotein (transmembrane) nmb	10457	-2,38

down	BRINP1	bone morphogenetic protein/retinoic acid inducible neural-specific 1	1620	-2,39
down	ICAM1	intercellular adhesion molecule 1	3383	-2,40
down	ABCA8	ATP-binding cassette, sub-family A (ABC1), member 8	10351	-2,40
down	IL1R1	interleukin 1 receptor, type I	3554	-2,41
down	SREBF1	sterol regulatory element binding transcription factor 1	6720	-2,42
down	SLC7A8	solute carrier family 7 (amino acid transporter light chain, L system), member 8	23428	-2,42
down	VCX3B	variable charge, X-linked 3B	425054	-2,42
down	STC1	stanniocalcin 1	6781	-2,44
down	APCDD1	adenomatosis polyposis coli down-regulated 1	147495	-2,45
down	HAUS7	HAUS augmin-like complex, subunit 7	55559	-2,46
down	PLXNC1	plexin C1	10154	-2,49
down	NRN1	neuritin 1	51299	-2,50
down	SCD	stearoyl-CoA desaturase (delta-9-desaturase)	6319	-2,51
down	RNPS1	RNA binding protein S1, serine-rich domain	10921	-2,52
down	OAF	OAF homolog (Drosophila)	220323	-2,53
down	CTSL	cathepsin L	1514	-2,54
down	SRSF7	serine/arginine-rich splicing factor 7	6432	-2,54
down	KCND2	potassium voltage-gated channel, Shal-related subfamily, member 2	3751	-2,56
down	FLJ38717	FLJ38717 protein	401261	-2,56
down	MAFB	v-maf avian musculoaponeurotic fibrosarcoma oncogene homolog B	9935	-2,59
down	CCL2	chemokine (C-C motif) ligand 2	6347	-2,63
down	SFRP1	secreted frizzled-related protein 1	6422	-2,64
down	PTGS2	prostaglandin-endoperoxide synthase 2 (prostaglandin G/H synthase and cyclooxygenase)	5743	-2,66
down	SEMA3A	sema domain, immunoglobulin domain (Ig), short basic domain, secreted, (semaphorin) 3A	10371	-2,66
down	MME	membrane metallo-endopeptidase	4311	-2,72
down	IFITM1	interferon induced transmembrane protein 1	8519	-2,72
down	CLU	clusterin	1191	-2,74
down	TNFSF10	tumor necrosis factor (ligand) superfamily, member 10	8743	-2,81
down	LCE2A	late cornified envelope 2A	353139	-2,86
down	HIST1H2BM	histone cluster 1, H2bm	8342	-2,87
down	FMOD	fibromodulin	2331	-2,94
down	KIT	v-kit Hardy-Zuckerman 4 feline sarcoma viral oncogene homolog	3815	-2,96
down	LRRC32	leucine rich repeat containing 32	2615	-2,99
down	MIR1247	microRNA 1247	100302145	-2,99
down	CCBE1	collagen and calcium binding EGF domains 1	147372	-2,99
down	ANGPTL2	angiopoietin-like 2	23452	-3,00

down	SERPINF1	serpin peptidase inhibitor, clade F (alpha-2 antiplasmin, pigment epithelium derived factor), member 1	5176	-3,09
down	C3	complement component 3	718	-3,14
down	PTGDS	prostaglandin D2 synthase 21kDa (brain)	5730	-3,16
down	AKAP12	A kinase (PRKA) anchor protein 12	9590	-3,16
down	KLF4	Kruppel-like factor 4 (gut)	9314	-3,28
down	MAP1LC3B2	microtubule-associated protein 1 light chain 3 beta 2	643246	-3,35
down	PDGFRL	platelet-derived growth factor receptor-like	5157	-3,36
down	ADH1B	alcohol dehydrogenase 1B (class I), beta polypeptide	125	-3,38
down	IL13RA2	interleukin 13 receptor, alpha 2	3598	-3,48
down	CDON	cell adhesion associated, oncogene regulated	50937	-3,57
down	PRELP	proline/arginine-rich end leucine-rich repeat protein	5549	-3,62
down	WISP2	WNT1 inducible signaling pathway protein 2	8839	-3,62
down	ABCA6	ATP-binding cassette, sub-family A (ABC1), member 6	23460	-3,74
down	RSPO3	R-spondin 3	84870	-4,14
down	COLEC12	collectin sub-family member 12	81035	-4,14
down	FOSB	FBJ murine osteosarcoma viral oncogene homolog B	2354	-4,16
down	ABCA9	ATP-binding cassette, sub-family A (ABC1), member 9	10350	-4,29
down	APOD	apolipoprotein D	347	-4,30
down	RCAN2	regulator of calcineurin 2	10231	-5,30
down	PDGFD	platelet derived growth factor D	80310	-5,37
down	MMP1	matrix metallopeptidase 1 (interstitial collagenase)	4312	-7,29
down	DPP4	dipeptidyl-peptidase 4	1803	-10,99

Table S2. Complete list of up- and down-regulated genes in C2 cells.

Direction in C2 cells	Gene symbol	Gene name	Entrez Gene ID	Fold change C2 vs C0
up	ANKRD1	ankyrin repeat domain 1 (cardiac muscle)	27063	15,34
up	ASPN	asporin	54829	14,50
up	ITGA8	integrin, alpha 8	8516	14,40
up	SRGN	serglycin	5552	9,67
up	MSTN	myostatin	2660	9,15
up	HAPLN3	hyaluronan and proteoglycan link protein 3	145864	8,98
up	COL11A1	collagen, type XI, alpha 1	1301	8,34
up	FLG	filaggrin	2312	8,23
up	SCUBE3	signal peptide, CUB domain, EGF-like 3	222663	7,56
up	LGR5	leucine-rich repeat containing G protein-coupled receptor 5	8549	7,30
up	TM4SF20	transmembrane 4 L six family member 20	79853	6,93
up	KRTAP2-3	keratin associated protein 2-3	730755	6,79
up	B3GALT2	UDP-Gal:betaGlcNAc beta 1,3-galactosyltransferase, polypeptide 2	8707	6,76
up	NOTCH3	notch 3	4854	6,55
up	OXTR	oxytocin receptor	5021	6,30
up	SLC7A5	solute carrier family 7 (amino acid transporter light chain, L system), member 5	8140	6,01
up	ELN	elastin	2006	5,88
up	VLDLR	very low density lipoprotein receptor	7436	5,72
up	COL4A1	collagen, type IV, alpha 1	1282	5,53
up	PHKA1	phosphorylase kinase, alpha 1 (muscle)	5255	5,34
up	KRTAP1-5	keratin associated protein 1-5	83895	5,24
up	HTR2A	5-hydroxytryptamine (serotonin) receptor 2A, G protein-coupled	3356	5,06
up	EDN1	endothelin 1	1906	5,04
up	MAMDC2	MAM domain containing 2	256691	5,01
up	GATA6	GATA binding protein 6	2627	4,78
up	BNIP3	BCL2/adenovirus E1B 19kDa interacting protein 3	664	4,61
up	TES	testis derived transcript (3 LIM domains)	26136	4,57
up	KCTD20	potassium channel tetramerization domain containing 20	222658	4,50
up	AKAP6	A kinase (PRKA) anchor protein 6	9472	4,42
up	PSAT1	phosphoserine aminotransferase 1	29968	4,33
up	ACAN	aggrecan	176	4,27
up	LUZP2	leucine zipper protein 2	338645	4,23
up	SLC2A1	solute carrier family 2 (facilitated glucose transporter), member 1	6513	4,19
up	SYNPO2	synaptopodin 2	171024	4,14
up	WFDC1	WAP four-disulfide core domain 1	58189	4,13
up	CNN1	calponin 1, basic, smooth muscle	1264	4,11

up	GPC4	glypican 4	2239	4,10
up	ITGA6	integrin, alpha 6	3655	4,05
up	MARCH4	membrane-associated ring finger (C3HC4) 4, E3 ubiquitin protein ligase	57574	4,05
up	RIMS1	regulating synaptic membrane exocytosis 1	22999	4,04
up	PPP1R14A	protein phosphatase 1, regulatory (inhibitor) subunit 14A	94274	4,02
up	RDH10	retinol dehydrogenase 10 (all-trans)	157506	4,02
up	FNDC1	fibronectin type III domain containing 1	84624	4,00
up	TNFSF4	tumor necrosis factor (ligand) superfamily, member 4	7292	3,94
up	PDE11A	phosphodiesterase 11A	50940	3,91
up	VEGFA	vascular endothelial growth factor A	7422	3,82
up	MYH11	myosin, heavy chain 11, smooth muscle	4629	3,82
up	LINC00312	long intergenic non-protein coding RNA 312	29931	3,74
up	GYS1	glycogen synthase 1 (muscle)	2997	3,72
up	CDH8	cadherin 8, type 2	1006	3,68
up	CHAC1	ChaC, cation transport regulator homolog 1 (E, coli)	79094	3,61
up	HUNK	hormonally up-regulated Neu-associated kinase	30811	3,60
up	MIR614	microRNA 614	693199	3,60
up	SOX9	SRY (sex determining region Y)-box 9	6662	3,57
up	WNT5A	wingless-type MMTV integration site family, member 5A	7474	3,53
up	PLOD2	procollagen-lysine, 2-oxoglutarate 5-dioxygenase 2	5352	3,53
up	LEP	leptin	3952	3,53
up	NUAK1	NUAK family, SNF1-like kinase, 1	9891	3,50
up	RGCC	regulator of cell cycle	28984	3,48
up	HSPB7	heat shock 27kDa protein family, member 7 (cardiovascular)	27129	3,42
up	CD24	CD24 molecule	100133941	3,41
up	PDCD1LG2	programmed cell death 1 ligand 2	80380	3,39
up	PGK1	phosphoglycerate kinase 1	5230	3,39
up	SLC16A3	solute carrier family 16 (monocarboxylate transporter), member 3	9123	3,38
up	KCNE4	potassium voltage-gated channel, Isk-related family, member 4	23704	3,37
up	LOXL2	lysyl oxidase-like 2	4017	3,35
up	SORBS2	sorbin and SH3 domain containing 2	8470	3,35
up	PLN	phospholamban	5350	3,35
up	IL20RB	interleukin 20 receptor beta	53833	3,30
up	SCN9A	sodium channel, voltage-gated, type IX, alpha subunit	6335	3,30
up	TSPAN18	tetraspanin 18	90139	3,29
up	CLIC3	chloride intracellular channel 3	9022	3,24
up	ITGA11	integrin, alpha 11	22801	3,24
up	DSP	desmoplakin	1832	3,14

up	DCLK2	doublecortin-like kinase 2	166614	3,14
up	WWC2	WW and C2 domain containing 2	80014	3,13
up	SEMA7A	semaphorin 7A, GPI membrane anchor (John Milton Hagen blood group)	8482	3,12
up	SNAI1	snail family zinc finger 1	6615	3,12
up	RASGRP1	RAS guanyl releasing protein 1 (calcium and DAG-regulated)	10125	3,11
up	TMEM45A	transmembrane protein 45A	55076	3,10
up	PDK1	pyruvate dehydrogenase kinase, isozyme 1	5163	3,10
up	PLCB4	phospholipase C, beta 4	5332	3,08
up	SLC16A4	solute carrier family 16, member 4	9122	3,08
up	ANGPTL4	angiopoietin-like 4	51129	3,04
up	EDIL3	EGF-like repeats and discoidin I-like domains 3	10085	3,03
up	PADI2	peptidyl arginine deiminase, type II	11240	3,02
up	FRY	furry homolog (Drosophila)	10129	3,02
up	PPP1R13L	protein phosphatase 1, regulatory subunit 13 like	10848	3,01
up	LPCAT2	lysophosphatidylcholine acyltransferase 2	54947	2,99
up	DACT1	dishevelled-binding antagonist of beta-catenin 1	51339	2,98
up	SORT1	sortilin 1	6272	2,98
up	TGM2	transglutaminase 2	7052	2,98
up	GRAMD3	GRAM domain containing 3	65983	2,98
up	IFFO2	intermediate filament family orphan 2	126917	2,96
up	NPAS2	neuronal PAS domain protein 2	4862	2,96
up	DIAPH3	diaphanous-related formin 3	81624	2,95
up	ASNS	asparagine synthetase (glutamine-hydrolyzing)	440	2,94
up	MRVI1	murine retrovirus integration site 1 homolog	10335	2,94
up	GLS	glutaminase	2744	2,93
up	LMCD1	LIM and cysteine-rich domains 1	29995	2,92
up	GPRC5A	G protein-coupled receptor, class C, group 5, member A	9052	2,92
up	NALCN	sodium leak channel, non-selective	259232	2,91
up	AVEN	apoptosis, caspase activation inhibitor	57099	2,90
up	GFRA1	GDNF family receptor alpha 1	2674	2,88
up	LGMN	legumain	5641	2,88
up	HSPG2	heparan sulfate proteoglycan 2	3339	2,85
up	ALDH1B1	aldehyde dehydrogenase 1 family, member B1	219	2,85
up	PSMD3	proteasome (prosome, macropain) 26S subunit, non-ATPase, 3	5709	2,80
up	MIR27B	microRNA 27b	407019	2,80
up	ZNF699	zinc finger protein 699	374879	2,79
up	HMCN1	hemicentin 1	83872	2,79
up	SFRP4	secreted frizzled-related protein 4	6424	2,78
up	COL4A2	collagen, type IV, alpha 2	1284	2,77
up	MYLK	myosin light chain kinase	4638	2,77

up	MFAP5	microfibrillar associated protein 5	8076	2,77
up	P4HA1	prolyl 4-hydroxylase, alpha polypeptide I	5033	2,75
up	F3	coagulation factor III (thromboplastin, tissue factor)	2152	2,74
up	CEBPG	CCAAT/enhancer binding protein (C/EBP), gamma	1054	2,73
up	RNF144B	ring finger protein 144B	255488	2,72
up	TUFT1	tuftelin 1	7286	2,71
up	HAPLN1	hyaluronan and proteoglycan link protein 1	1404	2,71
up	PDE5A	phosphodiesterase 5A, cGMP-specific	8654	2,69
up	OCLM	oculomedin	10896	2,69
up	SORBS1	sorbin and SH3 domain containing 1	10580	2,69
up	AMIGO2	adhesion molecule with Ig-like domain 2	347902	2,68
up	RP11-469A15,2	novel transcript	---	2,67
up	BOK	BCL2-related ovarian killer	666	2,66
up	BMP6	bone morphogenetic protein 6	654	2,65
up	PRPS1	phosphoribosyl pyrophosphate synthetase 1	5631	2,63
up	IVNS1ABP	influenza virus NS1A binding protein	10625	2,62
up	TEAD3	TEA domain family member 3	7005	2,62
up	MICAL2	microtubule associated monooxygenase, calponin and LIM domain containing 2	9645	2,62
up	STC1	stanniocalcin 1	6781	2,62
up	KRTAP1-1	keratin associated protein 1-1	81851	2,62
up	MALL	mal, T-cell differentiation protein-like	7851	2,62
up	ADIRF	adipogenesis regulatory factor	10974	2,61
up	ERRFI1	ERBB receptor feedback inhibitor 1	54206	2,61
up	KRT19	keratin 19	3880	2,61
up	SEL1L3	sel-1 suppressor of lin-12-like 3 (C, elegans)	23231	2,61
up	PKP4	plakophilin 4	8502	2,61
up	CPEB2	cytoplasmic polyadenylation element binding protein 2	132864	2,59
up	TSPAN2	tetraspanin 2	10100	2,59
up	LMOD1	leiomodin 1 (smooth muscle)	25802	2,58
up	PDLIM5	PDZ and LIM domain 5	10611	2,58
up	ADAMTS12	ADAM metallopeptidase with thrombospondin type 1 motif, 12	81792	2,55
up	BAIAP2L1	BAI1-associated protein 2-like 1	55971	2,55
up	SERPINE1	serpin peptidase inhibitor, clade E (nexin, plasminogen activator inhibitor type 1), member 1	5054	2,55
up	NEK7	NIMA-related kinase 7	140609	2,55
up	WNT2	wingless-type MMTV integration site family member 2	7472	2,54
up	MYH2	myosin, heavy chain 2, skeletal muscle, adult	4620	2,54
up	TGFBR1	transforming growth factor, beta receptor 1	7046	2,54
up	MFGE8	milk fat globule-EGF factor 8 protein	4240	2,53

up	MTHFD2	methylenetetrahydrofolate dehydrogenase (NADP+ dependent) 2, methenyltetrahydrofolate cyclohydrolase	10797	2,51
up	COL8A1	collagen, type VIII, alpha 1	1295	2,51
up	PTGIS	prostaglandin I2 (prostacyclin) synthase	5740	2,51
up	GPR155	G protein-coupled receptor 155	151556	2,50
up	ITGA1	integrin, alpha 1	3672	2,50
up	FLNB	filamin B, beta	2317	2,50
up	HAS2	hyaluronan synthase 2	3037	2,50
up	RASSF2	Ras association (RalGDS/AF-6) domain family member 2	9770	2,49
up	ADAMTS6	ADAM metallopeptidase with thrombospondin type 1 motif, 6	11174	2,48
up	C5orf28	chromosome 5 open reading frame 28	64417	2,47
up	RNF187	ring finger protein 187	149603	2,44
up	PLOD1	procollagen-lysine, 2-oxoglutarate 5-dioxygenase 1	5351	2,44
up	TRAM1	translocation associated membrane protein 1	23471	2,44
up	SSC5D	scavenger receptor cysteine rich domain containing (5 domains)	284297	2,43
up	CORO2B	coronin, actin binding protein, 2B	10391	2,42
up	CXorf56	chromosome X open reading frame 56	63932	2,42
up	JPH2	junctophilin 2	57158	2,42
up	KCNK6	potassium channel, subfamily K, member 6	9424	2,42
up	SASS6	spindle assembly 6 homolog (C, elegans)	163786	2,42
up	MCAM	melanoma cell adhesion molecule	4162	2,41
up	NFASC	neurofascin	23114	2,41
up	SDC2	syndecan 2	6383	2,40
up	ABCC3	ATP-binding cassette, sub-family C (CFTR/MRP), member 3	8714	2,40
up	WISP1	WNT1 inducible signaling pathway protein 1	8840	2,40
up	FLNC	filamin C, gamma	2318	2,39
up	NGF	nerve growth factor (beta polypeptide)	4803	2,39
up	TMEM130	transmembrane protein 130	222865	2,39
up	VASP	vasodilator-stimulated phosphoprotein	7408	2,39
up	JAM2	junctional adhesion molecule 2	58494	2,38
up	WIPF3	WAS/WASL interacting protein family, member 3	644150	2,38
up	RASA4	RAS p21 protein activator 4	10156	2,38
up	FAM101B	family with sequence similarity 101, member B	359845	2,38
up	ALDH1L2	aldehyde dehydrogenase 1 family, member L2	160428	2,38
up	POLR2J	polymerase (RNA) II (DNA directed) polypeptide J, 13,3kDa	5439	2,36
up	FOXG1	forkhead box G1	2290	2,35
up	ATP10A	ATPase, class V, type 10A	57194	2,35
up	MYH10	myosin, heavy chain 10, non-muscle	4628	2,35
up	PFKP	phosphofructokinase, platelet	5214	2,34

up	ERCC6	excision repair cross-complementation group 6	2074	2,33
up	SYTL2	synaptotagmin-like 2	54843	2,33
up	CEP112	centrosomal protein 112kDa	201134	2,33
up	FHOD3	formin homology 2 domain containing 3	80206	2,32
up	INHBB	inhibin, beta B	3625	2,32
up	ARSJ	arylsulfatase family, member J	79642	2,31
up	PMM2	phosphomannomutase 2	5373	2,31
up	RAPH1	Ras association (RalGDS/AF-6) and pleckstrin homology domains 1	65059	2,30
up	PACSIN3	protein kinase C and casein kinase substrate in neurons 3	29763	2,30
up	HSPA2	heat shock 70kDa protein 2	3306	2,29
up	CDKL5	cyclin-dependent kinase-like 5	6792	2,29
up	SLC4A4	solute carrier family 4 (sodium bicarbonate cotransporter), member 4	8671	2,29
up	PLCB1	phospholipase C, beta 1 (phosphoinositide-specific)	23236	2,28
up	UNC5B	unc-5 homolog B (C, elegans)	219699	2,27
up	SLC1A4	solute carrier family 1 (glutamate/neutral amino acid transporter), member 4	6509	2,27
up	ECM2	extracellular matrix protein 2, female organ and adipocyte specific	1842	2,26
up	FAM66C	family with sequence similarity 66, member C	440078	2,25
up	PPME1	protein phosphatase methylesterase 1	51400	2,25
up	ITGBL1	integrin, beta-like 1 (with EGF-like repeat domains)	9358	2,25
up	PDLIM7	PDZ and LIM domain 7 (enigma)	9260	2,24
up	RUSC2	RUN and SH3 domain containing 2	9853	2,24
up	WARS	tryptophanyl-tRNA synthetase	7453	2,24
up	CD55	CD55 molecule, decay accelerating factor for complement (Cromer blood group)	1604	2,24
up	CTGF	connective tissue growth factor	1490	2,23
up	PVR	poliovirus receptor	5817	2,23
up	YEATS2	YEATS domain containing 2	55689	2,23
up	LINC01023	long intergenic non-protein coding RNA 1023	100652853	2,21
up	RGS19	regulator of G-protein signaling 19	10287	2,20
up	TRIB3	tribbles pseudokinase 3	57761	2,20
up	MUC1	mucin 1, cell surface associated	4582	2,20
up	MEGF6	multiple EGF-like-domains 6	1953	2,20
up	A2M	alpha-2-macroglobulin	2	2,18
up	TSPAN13	tetraspanin 13	27075	2,18
up	FAT1	FAT atypical cadherin 1	2195	2,17
up	PCK2	phosphoenolpyruvate carboxykinase 2 (mitochondrial)	5106	2,17
up	IDH2	isocitrate dehydrogenase 2 (NADP+), mitochondrial	3418	2,16
up	SDPR	serum deprivation response	8436	2,16
up	PTPRB	protein tyrosine phosphatase, receptor type, B	5787	2,15

up	JAG1	jagged 1	182	2,15
up	COL5A1	collagen, type V, alpha 1	1289	2,15
up	COMP	cartilage oligomeric matrix protein	1311	2,14
up	ACTR1B	ARP1 actin-related protein 1 homolog B, centractin beta (yeast)	10120	2,14
up	AFF3	AF4/FMR2 family, member 3	3899	2,14
up	NEXN	nexilin (F actin binding protein)	91624	2,14
up	VCAN	versican	1462	2,14
up	NPR3	natriuretic peptide receptor 3	4883	2,13
up	RP11-23E10,2	novel transcript	---	2,13
up	INIP	INTS3 and NABP interacting protein	58493	2,12
up	INA	internexin neuronal intermediate filament protein, alpha	9118	2,12
up	USP53	ubiquitin specific peptidase 53	54532	2,12
up	MIR4640	microRNA 4640	100616237	2,12
up	GCLM	glutamate-cysteine ligase, modifier subunit	2730	2,11
up	GOLGA8A	golgin A8 family, member A	23015	2,11
up	C7orf60	chromosome 7 open reading frame 60	154743	2,11
up	FAM219A	family with sequence similarity 219, member A	203259	2,10
up	KIAA1462	KIAA1462	57608	2,10
up	SGPL1	sphingosine-1-phosphate lyase 1	8879	2,10
up	NEDD1	neural precursor cell expressed, developmentally down-regulated 1	121441	2,10
up	COL5A2	collagen, type V, alpha 2	1290	2,10
up	GCNT4	glucosaminyl (N-acetyl) transferase 4, core 2	51301	2,09
up	DAAM1	dishevelled associated activator of morphogenesis 1	23002	2,09
up	BAMBI	BMP and activin membrane-bound inhibitor	25805	2,09
up	SLC25A4	solute carrier family 25 (mitochondrial carrier; adenine nucleotide translocator), member 4	291	2,09
up	SGCD	sarcoglycan, delta (35kDa dystrophin-associated glycoprotein)	6444	2,09
up	SSH1	slingshot protein phosphatase 1	54434	2,09
up	C1orf198	chromosome 1 open reading frame 198	84886	2,09
up	TGFBR3L	transforming growth factor, beta receptor III-like	100507588	2,09
up	ARL4D	ADP-ribosylation factor-like 4D	379	2,08
up	HIAT1	hippocampus abundant transcript 1	64645	2,08
up	EIF4EBP1	eukaryotic translation initiation factor 4E binding protein 1	1978	2,08
up	TPM1	tropomyosin 1 (alpha)	7168	2,08
up	PKD1	polycystic kidney disease 1 (autosomal dominant)	5310	2,08
up	ENO1	enolase 1, (alpha)	2023	2,08
up	COL8A2	collagen, type VIII, alpha 2	1296	2,08
up	DHRS3	dehydrogenase/reductase (SDR family) member 3	9249	2,07
up	NLN	neurolysin (metallopeptidase M3 family)	57486	2,07

up	SLC1A5	solute carrier family 1 (neutral amino acid transporter), member 5	6510	2,07
up	CALD1	caldesmon 1	800	2,07
up	OR2T35	olfactory receptor, family 2, subfamily T, member 35	403244	2,07
up	LARGE	like-glycosyltransferase	9215	2,06
up	RNF181	ring finger protein 181	51255	2,06
up	TRAF5	TNF receptor-associated factor 5	7188	2,05
up	C19orf25	chromosome 19 open reading frame 25	148223	2,05
up	DDAH1	dimethylarginine dimethylaminohydrolase 1	23576	2,05
up	COL12A1	collagen, type XII, alpha 1	1303	2,05
up	SNORD19B	small nucleolar RNA, C/D box 19B	100113381	2,05
up	LYPD6B	LY6/PLAUR domain containing 6B	130576	2,04
up	PCGF5	polycomb group ring finger 5	84333	2,03
up	ROR1	receptor tyrosine kinase-like orphan receptor 1	4919	2,03
up	TECPR2	tectonin beta-propeller repeat containing 2	9895	2,03
up	JADE1	jade family PHD finger 1	79960	2,03
up	ENTPD4	ectonucleoside triphosphate diphosphohydrolase 4	9583	2,02
up	MEG3	maternally expressed 3 (non-protein coding)	55384	2,02
up	IMPA2	inositol(myo)-1(or 4)-monophosphatase 2	3613	2,02
up	RNF217	ring finger protein 217	154214	2,02
up	TTLL7	tubulin tyrosine ligase-like family, member 7	79739	2,01
up	IGFBP5	insulin-like growth factor binding protein 5	3488	2,01
up	MKNK2	MAP kinase interacting serine/threonine kinase 2	2872	2,01
up	SLC8A1	solute carrier family 8 (sodium/calcium exchanger), member 1	6546	2,01
up	TUBE1	tubulin, epsilon 1	51175	2,01
up	SLC38A4	solute carrier family 38, member 4	55089	2,01
up	RAVER2	ribonucleoprotein, PTB-binding 2	55225	2,00
down	IFIT3	interferon-induced protein with tetratricopeptide repeats 3	3437	-2,01
down	PPAP2B	phosphatidic acid phosphatase type 2B	8613	-2,01
down	USP17L23	ubiquitin specific peptidase 17-like family member 23	---	-2,01
down	DHCR24	24-dehydrocholesterol reductase	1718	-2,02
down	CTAGE1	cutaneous T-cell lymphoma-associated antigen 1	64693	-2,03
down	ZNF563	zinc finger protein 563	147837	-2,03
down	IL1R1	interleukin 1 receptor, type I	3554	-2,03
down	EEPD1	endonuclease/exonuclease/phosphatase family domain containing 1	80820	-2,03
down	PHLDA1	pleckstrin homology-like domain, family A, member 1	22822	-2,04
down	NAV2	neuron navigator 2	89797	-2,05
down	MCL1	myeloid cell leukemia 1	4170	-2,05
down	ZFP36	ZFP36 ring finger protein	7538	-2,05

down	ALDH3A2	aldehyde dehydrogenase 3 family, member A2	224	-2,06
down	SAT1	spermidine/spermine N1-acetyltransferase 1	6303	-2,06
down	ARMC9	armadillo repeat containing 9	80210	-2,07
down	CLIC2	chloride intracellular channel 2	1193	-2,07
down	OR2L3	olfactory receptor, family 2, subfamily L, member 3	391192	-2,10
down	MYLIP	myosin regulatory light chain interacting protein	29116	-2,11
down	TMTC1	transmembrane and tetratricopeptide repeat containing 1	83857	-2,11
down	SLC1A3	solute carrier family 1 (glial high affinity glutamate transporter), member 3	6507	-2,11
down	IFI16	interferon, gamma-inducible protein 16	3428	-2,12
down	PTGFR	prostaglandin F receptor (FP)	5737	-2,12
down	HSPA4L	heat shock 70kDa protein 4-like	22824	-2,13
down	GLUD1	glutamate dehydrogenase 1	2746	-2,14
down	IGSF10	immunoglobulin superfamily, member 10	285313	-2,14
down	SLC40A1	solute carrier family 40 (iron-regulated transporter), member 1	30061	-2,14
down	KLF6	Kruppel-like factor 6	1316	-2,14
down	DNM1	dynamin 1	1759	-2,15
down	PROS1	protein S (alpha)	5627	-2,15
down	FASN	fatty acid synthase	2194	-2,16
down	MME	membrane metallo-endopeptidase	4311	-2,16
down	HCG9	HLA complex group 9 (non-protein coding)	10255	-2,16
down	PTGDS	prostaglandin D2 synthase 21kDa (brain)	5730	-2,16
down	SNORA70	small nucleolar RNA, H/ACA box 70	26778	-2,18
down	ZCCHC14	zinc finger, CCHC domain containing 14	23174	-2,18
down	LINC01002	long intergenic non-protein coding RNA 1002	399844	-2,18
down	HIST1H2BM	histone cluster 1, H2bm	8342	-2,18
down	IFITM1	interferon induced transmembrane protein 1	8519	-2,21
down	DAB1	Dab, reelin signal transducer, homolog 1 (Drosophila)	1600	-2,23
down	SCPEP1	serine carboxypeptidase 1	59342	-2,23
down	SAMHD1	SAM domain and HD domain 1	25939	-2,24
down	DCN	decorin	1634	-2,25
down	ZNF480	zinc finger protein 480	147657	-2,26
down	EGFR	epidermal growth factor receptor	1956	-2,27
down	TGFBR3	transforming growth factor, beta receptor III	7049	-2,31
down	SLC9A9	solute carrier family 9, subfamily A (NHE9, cation proton antiporter 9), member 9	285195	-2,32
down	ZNF608	zinc finger protein 608	57507	-2,34
down	TNXB	tenascin XB	7148	-2,34
down	UBC	ubiquitin C	7316	-2,35
down	KCND2	potassium voltage-gated channel, Shal-related subfamily, member 2	3751	-2,37
down	KIF4B	kinesin family member 4B	285643	-2,37

down	HIST2H2AB	histone cluster 2, H2ab	317772	-2,38
down	TBC1D29	TBC1 domain family, member 29	26083	-2,40
down	SOX4	SRY (sex determining region Y)-box 4	6659	-2,41
down	ARMCX1	armadillo repeat containing, X-linked 1	51309	-2,42
down	OSR2	odd-skipped related transciption factor 2	116039	-2,44
down	FADS1	fatty acid desaturase 1	3992	-2,44
down	CLU	clusterin	1191	-2,48
down	OR56A5	olfactory receptor, family 56, subfamily A, member 5	390084	-2,49
down	SOCS3	suppressor of cytokine signaling 3	9021	-2,51
down	OR2A14	olfactory receptor, family 2, subfamily A, member 14	135941	-2,52
down	GH2	growth hormone 2	2689	-2,53
down	CDON	cell adhesion associated, oncogene regulated	50937	-2,54
down	SRSF7	serine/arginine-rich splicing factor 7	6432	-2,55
down	EGR1	early growth response 1	1958	-2,60
down	IER2	immediate early response 2	9592	-2,60
down	PPARGC1A	peroxisome proliferator-activated receptor gamma, coactivator 1 alpha	10891	-2,60
down	AKAP12	A kinase (PRKA) anchor protein 12	9590	-2,65
down	PLXNC1	plexin C1	10154	-2,65
down	PDE7B	phosphodiesterase 7B	27115	-2,67
down	TAF9B	TAF9B RNA polymerase II, TATA box binding protein (TBP)-associated factor, 31kDa	51616	-2,74
down	TCEB3C	transcription elongation factor B polypeptide 3C (elongin A3)	162699	-2,74
down	JUNB	jun B proto-oncogene	3726	-2,75
down	SLC43A3	solute carrier family 43, member 3	29015	-2,76
down	SCARA3	scavenger receptor class A, member 3	51435	-2,77
down	RRP7B	ribosomal RNA processing 7 homolog B (S, cerevisiae)	91695	-2,77
down	LGALS9B	lectin, galactoside-binding, soluble, 9B	284194	-2,77
down	TNFSF10	tumor necrosis factor (ligand) superfamily, member 10	8743	-2,81
down	INSIG1	insulin induced gene 1	3638	-2,84
down	ABCC9	ATP-binding cassette, sub-family C (CFTR/MRP), member 9	10060	-2,85
down	MIR3620	microRNA 3620	100500810	-2,88
down	DBET	D4Z4 binding element transcript (non-protein coding)	100419743	-2,95
down	NFKBIZ	nuclear factor of kappa light polypeptide gene enhancer in B-cells inhibitor, zeta	64332	-2,98
down	JUN	jun proto-oncogene	3725	-3,00
down	SERPINF1	serpin peptidase inhibitor, clade F (alpha-2 antiplasmin, pigment epithelium derived factor), member 1	5176	-3,04
down	ANGPTL2	angiopoietin-like 2	23452	-3,05
down	PDGFD	platelet derived growth factor D	80310	-3,07

down	SLC7A8	solute carrier family 7 (amino acid transporter light chain, L system), member 8	23428	-3,08
down	MAFB	v-maf avian musculoaponeurotic fibrosarcoma oncogene homolog B	9935	-3,15
down	APCDD1	adenomatosis polyposis coli down-regulated 1	147495	-3,17
down	CDC25B	cell division cycle 25B	994	-3,19
down	PTGS2	prostaglandin-endoperoxide synthase 2 (prostaglandin G/H synthase and cyclooxygenase)	5743	-3,23
down	BRINP1	bone morphogenetic protein/retinoic acid inducible neural-specific 1	1620	-3,26
down	KLF4	Kruppel-like factor 4 (gut)	9314	-3,28
down	DUX4L1	double homeobox 4 like 1	22947	-3,31
down	PDGFRL	platelet-derived growth factor receptor-like	5157	-3,36
down	PCSK7	proprotein convertase subtilisin/kexin type 7	9159	-3,37
down	FLT3LG	fms-related tyrosine kinase 3 ligand	2323	-3,44
down	LDLR	low density lipoprotein receptor	3949	-3,50
down	USP17L15	ubiquitin specific peptidase 17-like family member 15	100288520	-3,54
down	LRRC32	leucine rich repeat containing 32	2615	-3,58
down	GPNMB	glycoprotein (transmembrane) nmb	10457	-3,60
down	ZNF595	zinc finger protein 595	152687	-3,60
down	OC90	otoconin 90	729330	-3,60
down	RCAN2	regulator of calcineurin 2	10231	-3,62
down	FOS	FBJ murine osteosarcoma viral oncogene homolog	2353	-3,79
down	COLEC12	collectin sub-family member 12	81035	-3,81
down	SCD	stearoyl-CoA desaturase (delta-9-desaturase)	6319	-3,84
down	WISP2	WNT1 inducible signaling pathway protein 2	8839	-3,85
down	IL13RA2	interleukin 13 receptor, alpha 2	3598	-4,01
down	GALNT15	polypeptide N-acetylgalactosaminyltransferase 15	117248	-4,12
down	ADH1B	alcohol dehydrogenase 1B (class I), beta polypeptide	125	-4,27
down	ABCA9	ATP-binding cassette, sub-family A (ABC1), member 9	10350	-4,29
down	APOD	apolipoprotein D	347	-4,30
down	DUX4	double homeobox 4	100288687	-4,39
down	DPP4	dipeptidyl-peptidase 4	1803	-4,45
down	MMP3	matrix metallopeptidase 3 (stromelysin 1, progelatinase)	4314	-5,07
down	USP17L5	ubiquitin specific peptidase 17-like family member 5	728386	-5,56
down	ABCA6	ATP-binding cassette, sub-family A (ABC1), member 6	23460	-5,60
down	RSPO3	R-spondin 3	84870	-6,83
down	CTSK	cathepsin K	1513	-7,29
down	MMP1	matrix metallopeptidase 1 (interstitial collagenase)	4312	-7,96
down	FOSB	FBJ murine osteosarcoma viral oncogene homolog B	2354	-8,42

Table S3. Complete list of up- and down-regulated genes in C3 cells.

Direction in C3 cells	Genesymbol	Gene name	Entrez Gene ID	Fold change C3 vs C0
up	KRTAP2-3	keratin associated protein 2-3	730755	12,81
up	SCUBE3	signal peptide, CUB domain, EGF-like 3	222663	10,53
up	ANKRD1	ankyrin repeat domain 1 (cardiac muscle)	27063	9,15
up	OXTR	oxytocin receptor	5021	6,97
up	EDN1	endothelin 1	1906	5,99
up	SRGN	serglycin	5552	5,93
up	KRTAP1-5	keratin associated protein 1-5	83895	5,92
up	ANLN	anillin, actin binding protein	54443	5,85
up	FBN2	fibrillin 2	2201	5,27
up	PRR11	proline rich 11	55771	5,18
up	KIF20A	kinesin family member 20A	10112	5,06
up	MYPN	myopalladin	84665	4,87
up	ADIRF	adipogenesis regulatory factor	10974	4,79
up	ARSJ	arylsulfatase family, member J	79642	4,67
up	HAPLN3	hyaluronan and proteoglycan link protein 3	145864	4,58
up	NGF	nerve growth factor (beta polypeptide)	4803	4,47
up	BUB1	BUB1 mitotic checkpoint serine/threonine kinase	699	4,42
up	TPX2	TPX2, microtubule-associated	22974	4,40
up	MKI67	marker of proliferation Ki-67	4288	4,37
up	CEP55	centrosomal protein 55kDa	55165	4,36
up	KRT19	keratin 19	3880	4,24
up	TM4SF1	transmembrane 4 L six family member 1	4071	4,20
up	DCLK2	doublecortin-like kinase 2	166614	4,12
up	MARCH4	membrane-associated ring finger (C3HC4) 4, E3 ubiquitin protein ligase	57574	4,08
up	NOTCH3	notch 3	4854	4,05
up	COL4A1	collagen, type IV, alpha 1	1282	3,90
up	ELN	elastin	2006	3,82
up	DIAPH3	diaphanous-related formin 3	81624	3,77
up	SPAG5	sperm associated antigen 5	10615	3,72
up	SLC16A4	solute carrier family 16, member 4	9122	3,63
up	DLGAP5	discs, large (Drosophila) homolog-associated protein 5	9787	3,62
up	CCNA2	cyclin A2	890	3,59
up	PLK1	polo-like kinase 1	5347	3,57
up	MALL	mal, T-cell differentiation protein-like	7851	3,56
up	ITGA8	integrin, alpha 8	8516	3,55
up	SHCBP1	SHC SH2-domain binding protein 1	79801	3,55
up	SLC7A5	solute carrier family 7 (amino acid transporter light chain, L system), member 5	8140	3,51

up	SNAI1	snail family zinc finger 1	6615	3,36
up	PRC1	protein regulator of cytokinesis 1	9055	3,28
up	ASPM	asp (abnormal spindle) homolog, microcephaly associated (Drosophila)	259266	3,20
up	CDC20	cell division cycle 20	991	3,19
up	LINC00152	long intergenic non-protein coding RNA 152	112597	3,16
up	TOP2A	topoisomerase (DNA) II alpha 170kDa	7153	3,12
up	DSP	desmoplakin	1832	3,11
up	KIF11	kinesin family member 11	3832	3,11
up	PDCD1LG2	programmed cell death 1 ligand 2	80380	3,10
up	CENPE	centromere protein E, 312kDa	1062	3,09
up	C19orf25	chromosome 19 open reading frame 25	148223	3,06
up	SLC16A3	solute carrier family 16 (monocarboxylate transporter), member 3	9123	3,05
up	IFFO2	intermediate filament family orphan 2	126917	3,04
up	FOXC2	forkhead box C2 (MFH-1, mesenchyme forkhead 1)	2303	3,03
up	KIF14	kinesin family member 14	9928	2,98
up	NCAPG	non-SMC condensin I complex, subunit G	64151	2,94
up	ADAMTS6	ADAM metallopeptidase with thrombospondin type 1 motif, 6	11174	2,92
up	SEMA7A	semaphorin 7A, GPI membrane anchor (John Milton Hagen blood group)	8482	2,90
up	LOXL2	lysyl oxidase-like 2	4017	2,89
up	CDH2	cadherin 2, type 1, N-cadherin (neuronal)	1000	2,87
up	DLX2	distal-less homeobox 2	1746	2,87
up	DKK1	dickkopf WNT signaling pathway inhibitor 1	22943	2,83
up	SPOCD1	SPOC domain containing 1	90853	2,83
up	BIRC5	baculoviral IAP repeat containing 5	332	2,83
up	NETO2	neuropilin (NRP) and tolloid (TLL)-like 2	81831	2,79
up	BNIP3	BCL2/adenovirus E1B 19kDa interacting protein 3	664	2,78
up	HSPB7	heat shock 27kDa protein family, member 7 (cardiovascular)	27129	2,76
up	MSTN	myostatin	2660	2,76
up	CDA	cytidine deaminase	978	2,76
up	ZNF185	zinc finger protein 185 (LIM domain)	7739	2,74
up	TK1	thymidine kinase 1, soluble	7083	2,74
up	FAM101B	family with sequence similarity 101, member B	359845	2,74
up	ARHGAP11A	Rho GTPase activating protein 11A	9824	2,72
up	OCLM	oculomedin	10896	2,71
up	PFKP	phosphofructokinase, platelet	5214	2,70
up	B3GALT2	UDP-Gal:betaGlcNAc beta 1,3-galactosyltransferase, polypeptide 2	8707	2,69
up	CD24	CD24 molecule	100133941	2,69
up	TSPAN18	tetraspanin 18	90139	2,68

up	CHRM2	cholinergic receptor, muscarinic 2	1129	2,67
up	CASC5	cancer susceptibility candidate 5	57082	2,67
up	SPDL1	spindle apparatus coiled-coil protein 1	54908	2,65
up	NUSAP1	nucleolar and spindle associated protein 1	51203	2,64
up	CNN1	calponin 1, basic, smooth muscle	1264	2,64
up	BLID	BH3-like motif containing, cell death inducer	414899	2,63
up	CXorf56	chromosome X open reading frame 56	63932	2,62
up	ADAM23	ADAM metallopeptidase domain 23	8745	2,62
up	CDH8	cadherin 8, type 2	1006	2,61
up	TNFRSF12A	tumor necrosis factor receptor superfamily, member 12A	51330	2,60
up	PEG10	paternally expressed 10	23089	2,58
up	KIAA1524	KIAA1524	57650	2,55
up	LMO7DN	LMO7 downstream neighbor	729420	2,53
up	SCFD2	sec1 family domain containing 2	152579	2,51
up	ANKRD54	ankyrin repeat domain 54	129138	2,51
up	SLC8A1	solute carrier family 8 (sodium/calcium exchanger), member 1	6546	2,50
up	SMS	spermine synthase	6611	2,49
up	ARNTL2	aryl hydrocarbon receptor nuclear translocator-like 2	56938	2,48
up	WWC2	WW and C2 domain containing 2	80014	2,47
up	SLC17A9	solute carrier family 17 (vesicular nucleotide transporter), member 9	63910	2,47
up	LYPD6B	LY6/PLAUR domain containing 6B	130576	2,46
up	PRPS1	phosphoribosyl pyrophosphate synthetase 1	5631	2,45
up	TGFBI	transforming growth factor, beta-induced, 68kDa	7045	2,44
up	GYS1	glycogen synthase 1 (muscle)	2997	2,43
up	SSC5D	scavenger receptor cysteine rich domain containing (5 domains)	284297	2,43
up	FOXL1	forkhead box L1	2300	2,43
up	FOXM1	forkhead box M1	2305	2,43
up	MIR197	microRNA 197	406974	2,42
up	RASA4	RAS p21 protein activator 4	10156	2,41
up	BHLHE40	basic helix-loop-helix family, member e40	8553	2,41
up	LGMN	legumain	5641	2,40
up	SLC7A1	solute carrier family 7 (cationic amino acid transporter, y+ system), member 1	6541	2,40
up	FANCD2	Fanconi anemia, complementation group D2	2177	2,39
up	LPXN	leupaxin	9404	2,37
up	KIF18A	kinesin family member 18A	81930	2,37
up	IQGAP3	IQ motif containing GTPase activating protein 3	128239	2,37
up	COTL1	coactosin-like F-actin binding protein 1	23406	2,36
up	TSPAN13	tetraspanin 13	27075	2,35
up	SERTAD2	SERTA domain containing 2	9792	2,35

up	GATA6	GATA binding protein 6	2627	2,34
up	NLN	neurolysin (metallopeptidase M3 family)	57486	2,34
up	CDK1	cyclin-dependent kinase 1	983	2,33
up	SND1-IT1	SND1 intronic transcript 1 (non-protein coding)	27099	2,33
up	UCHL1	ubiquitin carboxyl-terminal esterase L1 (ubiquitin thiolesterase)	7345	2,33
up	PDE1C	phosphodiesterase 1C, calmodulin-dependent 70kDa	5137	2,33
up	TES	testis derived transcript (3 LIM domains)	26136	2,33
up	LGR5	leucine-rich repeat containing G protein-coupled receptor 5	8549	2,32
up	KCNE4	potassium voltage-gated channel, Isk-related family, member 4	23704	2,32
up	GLIPR1	GLI pathogenesis-related 1	11010	2,31
up	CGB8	chorionic gonadotropin, beta polypeptide 8	94115	2,31
up	HERC4	HECT and RLD domain containing E3 ubiquitin protein ligase 4	26091	2,31
up	GFRA1	GDNF family receptor alpha 1	2674	2,31
up	PDLIM5	PDZ and LIM domain 5	10611	2,30
up	TRAJ46	T cell receptor alpha joining 46	---	2,29
up	PRUNE2	prune homolog 2 (Drosophila)	158471	2,29
up	ITGA3	integrin, alpha 3 (antigen CD49C, alpha 3 subunit of VLA-3 receptor)	3675	2,28
up	ENTPD4	ectonucleoside triphosphate diphosphohydrolase 4	9583	2,28
up	LMNB1	lamin B1	4001	2,27
up	EIF4EBP1	eukaryotic translation initiation factor 4E binding protein 1	1978	2,26
up	TRIB1	tribbles pseudokinase 1	10221	2,26
up	ITGA6	integrin, alpha 6	3655	2,25
up	DEPDC1	DEP domain containing 1	55635	2,24
up	PSMD3	proteasome (prosome, macropain) 26S subunit, non-ATPase, 3	5709	2,23
up	SERPINE1	serpin peptidase inhibitor, clade E (nexin, plasminogen activator inhibitor type 1), member 1	5054	2,23
up	FLJ21369	uncharacterized protein FLJ21369	79860	2,23
up	LINC00862	long intergenic non-protein coding RNA 862	554279	2,22
up	PLOD2	procollagen-lysine, 2-oxoglutarate 5-dioxygenase 2	5352	2,22
up	PDE5A	phosphodiesterase 5A, cGMP-specific	8654	2,22
up	MMP3	matrix metallopeptidase 3 (stromelysin 1, progelatinase)	4314	2,22
up	CLIC3	chloride intracellular channel 3	9022	2,21
up	P4HA1	prolyl 4-hydroxylase, alpha polypeptide I	5033	2,20
up	PDLIM7	PDZ and LIM domain 7 (enigma)	9260	2,20
up	ACTN1	actinin, alpha 1	87	2,20
up	PLEKHA2	pleckstrin homology domain containing, family A (phosphoinositide binding specific) member 2	59339	2,20

up	PSAT1	phosphoserine aminotransferase 1	29968	2,20
up	ARL4D	ADP-ribosylation factor-like 4D	379	2,19
up	HSPB3	heat shock 27kDa protein 3	8988	2,19
up	PEAR1	platelet endothelial aggregation receptor 1	375033	2,19
up	SOX9	SRY (sex determining region Y)-box 9	6662	2,19
up	PDK1	pyruvate dehydrogenase kinase, isozyme 1	5163	2,19
up	HBEGF	heparin-binding EGF-like growth factor	1839	2,19
up	FMN2	formin 2	56776	2,19
up	TPM1	tropomyosin 1 (alpha)	7168	2,18
up	KCTD20	potassium channel tetramerization domain containing 20	222658	2,17
up	TMSB10	thymosin beta 10	9168	2,17
up	SH2D4A	SH2 domain containing 4A	63898	2,17
up	CRLF1	cytokine receptor-like factor 1	9244	2,17
up	GPRC5A	G protein-coupled receptor, class C, group 5, member A	9052	2,17
up	FLNC	filamin C, gamma	2318	2,16
up	KIAA0101	KIAA0101	9768	2,16
up	ASPN	asporin	54829	2,16
up	SLC2A1	solute carrier family 2 (facilitated glucose transporter), member 1	6513	2,16
up	MIR614	microRNA 614	693199	2,15
up	DCBLD1	discoidin, CUB and LCCL domain containing 1	285761	2,15
up	HSPG2	heparan sulfate proteoglycan 2	3339	2,14
up	CSRP1	cysteine and glycine-rich protein 1	1465	2,14
up	AVEN	apoptosis, caspase activation inhibitor	57099	2,14
up	VEGFC	vascular endothelial growth factor C	7424	2,14
up	SORT1	sortilin 1	6272	2,14
up	CKAP2	cytoskeleton associated protein 2	26586	2,13
up	SMYD2	SET and MYND domain containing 2	56950	2,13
up	GSTA1	glutathione S-transferase alpha 1	2938	2,13
up	KCNK6	potassium channel, subfamily K, member 6	9424	2,13
up	VAT1L	vesicle amine transport 1-like	57687	2,13
up	TAF1D	TATA box binding protein (TBP)-associated factor, RNA polymerase I, D, 41kDa	79101	2,12
up	RP11-469A15,2	novel transcript	---	2,12
up	FGF5	fibroblast growth factor 5	2250	2,12
up	HAPLN1	hyaluronan and proteoglycan link protein 1	1404	2,11
up	FLG	filaggrin	2312	2,11
up	FKBP11	FK506 binding protein 11, 19 kDa	51303	2,11
up	GGT2	gamma-glutamyltransferase 2	728441	2,11
up	CORO1C	coronin, actin binding protein, 1C	23603	2,11
up	AKAP6	A kinase (PRKA) anchor protein 6	9472	2,10

up	F3	coagulation factor III (thromboplastin, tissue factor)	2152	2,08
up	PRRX2	paired related homeobox 2	51450	2,07
up	SEL1L3	sel-1 suppressor of lin-12-like 3 (C, elegans)	23231	2,07
up	MICAL2	microtubule associated monooxygenase, calponin and LIM domain containing 2	9645	2,07
up	PLCB1	phospholipase C, beta 1 (phosphoinositide-specific)	23236	2,06
up	NPAS2	neuronal PAS domain protein 2	4862	2,05
up	LY6K	lymphocyte antigen 6 complex, locus K	54742	2,05
up	CCNB1	cyclin B1	891	2,05
up	C12orf75	chromosome 12 open reading frame 75	387882	2,05
up	MYBL1	v-myb avian myeloblastosis viral oncogene homolog-like 1	4603	2,05
up	XYLT1	xylosyltransferase I	64131	2,04
up	ABCC3	ATP-binding cassette, sub-family C (CFTR/MRP), member 3	8714	2,04
up	CKAP2L	cytoskeleton associated protein 2-like	150468	2,03
up	PMM2	phosphomannomutase 2	5373	2,03
up	LPCAT2	lysophosphatidylcholine acyltransferase 2	54947	2,02
up	ALCAM	activated leukocyte cell adhesion molecule	214	2,02
up	ASB6	ankyrin repeat and SOCS box containing 6	140459	2,02
up	IGHJ3	immunoglobulin heavy joining 3	---	2,02
up	TMEM14A	transmembrane protein 14A	28978	2,02
up	SH3D21	SH3 domain containing 21	79729	2,02
up	SCIN	scinderin	85477	2,01
up	C6orf62	chromosome 6 open reading frame 62	81688	2,01
up	SLC1A4	solute carrier family 1 (glutamate/neutral amino acid transporter), member 4	6509	2,00
down	LCE1C	late cornified envelope 1C	353133	-2,00
down	DCLK1	doublecortin-like kinase 1	9201	-2,01
down	NPC2	Niemann-Pick disease, type C2	10577	-2,01
down	DKFZP434I0714	uncharacterized protein DKFZP434I0714	54553	-2,02
down	OR2A14	olfactory receptor, family 2, subfamily A, member 14	135941	-2,02
down	CCL2	chemokine (C-C motif) ligand 2	6347	-2,03
down	HERC2	HECT and RLD domain containing E3 ubiquitin protein ligase 2	8924	-2,03
down	PROS1	protein S (alpha)	5627	-2,04
down	AKAP12	A kinase (PRKA) anchor protein 12	9590	-2,05
down	CYBRD1	cytochrome b reductase 1	79901	-2,05
down	SNORD116-2	small nucleolar RNA, C/D box 116-2	100033414	-2,05
down	REV3L	REV3-like, polymerase (DNA directed), zeta, catalytic subunit	5980	-2,06
down	FADS1	fatty acid desaturase 1	3992	-2,07
down	AKR7L	aldo-keto reductase family 7-like	246181	-2,08

down	IL6ST	interleukin 6 signal transducer	3572	-2,08
down	ARHGAP12	Rho GTPase activating protein 12	94134	-2,09
down	RAB29	RAB29, member RAS oncogene family	8934	-2,10
down	TP53INP1	tumor protein p53 inducible nuclear protein 1	94241	-2,10
down	MIR21	microRNA 21	406991	-2,12
down	MCL1	myeloid cell leukemia 1	4170	-2,12
down	DBET	D4Z4 binding element transcript (non-protein coding)	100419743	-2,12
down	TPP1	tripeptidyl peptidase I	1200	-2,12
down	MTSS1L	metastasis suppressor 1-like	92154	-2,13
down	CLIC2	chloride intracellular channel 2	1193	-2,14
down	LSAMP	limbic system-associated membrane protein	4045	-2,14
down	DUX4	double homeobox 4	100288687	-2,14
down	IGSF10	immunoglobulin superfamily, member 10	285313	-2,14
down	SEMA3D	sema domain, immunoglobulin domain (Ig), short basic domain, secreted, (semaphorin) 3D	223117	-2,14
down	HEXB	hexosaminidase B (beta polypeptide)	3074	-2,15
down	TBC1D29	TBC1 domain family, member 29	26083	-2,16
down	SLC39A8	solute carrier family 39 (zinc transporter), member 8	64116	-2,17
down	KRTAP10-5	keratin associated protein 10-5	386680	-2,17
down	SNED1	sushi, nidogen and EGF-like domains 1	25992	-2,17
down	ZNF480	zinc finger protein 480	147657	-2,17
down	FAP	fibroblast activation protein, alpha	2191	-2,17
down	ZNF600	zinc finger protein 600	162966	-2,18
down	C3	complement component 3	718	-2,18
down	SVIL	supervillin	6840	-2,18
down	LRIG3	leucine-rich repeats and immunoglobulin-like domains 3	121227	-2,19
down	WLS	wntless Wnt ligand secretion mediator	79971	-2,19
down	SNX2	sorting nexin 2	6643	-2,20
down	MDM2	MDM2 proto-oncogene, E3 ubiquitin protein ligase	4193	-2,21
down	FAM20A	family with sequence similarity 20, member A	54757	-2,21
down	ACVR2A	activin A receptor, type IIA	92	-2,22
down	JUNB	jun B proto-oncogene	3726	-2,22
down	GALNT15	polypeptide N-acetylgalactosaminyltransferase 15	117248	-2,22
down	SLC7A8	solute carrier family 7 (amino acid transporter light chain, L system), member 8	23428	-2,23
down	GAA	glucosidase, alpha; acid	2548	-2,23
down	CLU	clusterin	1191	-2,24
down	TGFBR3	transforming growth factor, beta receptor III	7049	-2,24
down	DRAM1	DNA-damage regulated autophagy modulator 1	55332	-2,25
down	MIR3620	microRNA 3620	100500810	-2,26
down	CCNL1	cyclin L1	57018	-2,26

down	DCN	decorin	1634	-2,30
down	PPAP2B	phosphatidic acid phosphatase type 2B	8613	-2,34
down	UBC	ubiquitin C	7316	-2,35
down	SNORA70	small nucleolar RNA, H/ACA box 70	26778	-2,36
down	IGKV1-16	immunoglobulin kappa variable 1-16	---	-2,37
down	LGALS9B	lectin, galactoside-binding, soluble, 9B	284194	-2,39
down	THBS2	thrombospondin 2	7058	-2,42
down	SERPING1	serpin peptidase inhibitor, clade G (C1 inhibitor), member 1	710	-2,43
down	C1R	complement component 1, r subcomponent	715	-2,44
down	CACNG8	calcium channel, voltage-dependent, gamma subunit 8	59283	-2,44
down	APCDD1	adenomatosis polyposis coli down-regulated 1	147495	-2,45
down	SCPEP1	serine carboxypeptidase 1	59342	-2,49
down	ZNF836	zinc finger protein 836	162962	-2,49
down	ZNF563	zinc finger protein 563	147837	-2,50
down	GAS1	growth arrest-specific 1	2619	-2,51
down	OR56A5	olfactory receptor, family 56, subfamily A, member 5	390084	-2,52
down	ALDH3A2	aldehyde dehydrogenase 3 family, member A2	224	-2,54
down	PCDHB7	protocadherin beta 7	56129	-2,54
down	SRSF7	serine/arginine-rich splicing factor 7	6432	-2,54
down	IFITM1	interferon induced transmembrane protein 1	8519	-2,56
down	ZMYM6NB	ZMYM6 neighbor	100506144	-2,56
down	OSR2	odd-skipped related transciption factor 2	116039	-2,56
down	SAMHD1	SAM domain and HD domain 1	25939	-2,59
down	MAFB	v-maf avian musculoaponeurotic fibrosarcoma oncogene homolog B	9935	-2,59
down	SVEP1	sushi, von Willebrand factor type A, EGF and pentraxin domain containing 1	79987	-2,59
down	SNORA72	small nucleolar RNA, H/ACA box 72	26775	-2,60
down	FLT3LG	fms-related tyrosine kinase 3 ligand	2323	-2,61
down	ABCC9	ATP-binding cassette, sub-family C (CFTR/MRP), member 9	10060	-2,63
down	SNORA11	small nucleolar RNA, H/ACA box 11	677799	-2,66
down	PPARGC1A	peroxisome proliferator-activated receptor gamma, coactivator 1 alpha	10891	-2,67
down	SLC1A3	solute carrier family 1 (glial high affinity glutamate transporter), member 3	6507	-2,69
down	SLC9A9	solute carrier family 9, subfamily A (NHE9, cation proton antiporter 9), member 9	285195	-2,76
down	KIT	v-kit Hardy-Zuckerman 4 feline sarcoma viral oncogene homolog	3815	-2,78
down	TNFSF10	tumor necrosis factor (ligand) superfamily, member 10	8743	-2,81
down	SFRP2	secreted frizzled-related protein 2	6423	-2,83
down	PTGS2	prostaglandin-endoperoxide synthase 2 (prostaglandin G/H synthase and cyclooxygenase)	5743	-2,85

down	ADM	adrenomedullin	133	-2,92
down	KCND2	potassium voltage-gated channel, Shal-related subfamily, member 2	3751	-2,99
down	ICAM1	intercellular adhesion molecule 1	3383	-3,00
down	SOX4	SRY (sex determining region Y)-box 4	6659	-3,00
down	CTNS	cystinosin, lysosomal cystine transporter	1497	-3,07
down	MYO1D	myosin ID	4642	-3,08
down	MFAP4	microfibrillar-associated protein 4	4239	-3,08
down	RRP7B	ribosomal RNA processing 7 homolog B (S, cerevisiae)	91695	-3,12
down	IL1R1	interleukin 1 receptor, type I	3554	-3,15
down	ZNF608	zinc finger protein 608	57507	-3,18
down	SMPDL3A	sphingomyelin phosphodiesterase, acid-like 3A	10924	-3,18
down	LRRC32	leucine rich repeat containing 32	2615	-3,20
down	PDPN	podoplanin	10630	-3,20
down	ADH1B	alcohol dehydrogenase 1B (class I), beta polypeptide	125	-3,22
down	PDGFRL	platelet-derived growth factor receptor-like	5157	-3,36
down	CDON	cell adhesion associated, oncogene regulated	50937	-3,39
down	C1S	complement component 1, s subcomponent	716	-3,41
down	KLF4	Kruppel-like factor 4 (gut)	9314	-3,50
down	FBLN1	fibulin 1	2192	-3,54
down	PRELP	proline/arginine-rich end leucine-rich repeat protein	5549	-3,55
down	IL13RA2	interleukin 13 receptor, alpha 2	3598	-3,61
down	ABCA8	ATP-binding cassette, sub-family A (ABC1), member 8	10351	-3,63
down	FIBIN	fin bud initiation factor homolog (zebrafish)	387758	-3,67
down	PLXNC1	plexin C1	10154	-3,76
down	SECTM1	secreted and transmembrane 1	6398	-3,78
down	JUN	jun proto-oncogene	3725	-3,86
down	GPNMB	glycoprotein (transmembrane) nmb	10457	-3,86
down	HSPA4L	heat shock 70kDa protein 4-like	22824	-3,90
down	SULF2	sulfatase 2	55959	-3,98
down	PAPPA	pregnancy-associated plasma protein A, pappalysin 1	5069	-4,00
down	SNORD116-14	small nucleolar RNA, C/D box 116-14	100033426	-4,20
down	FMOD	fibromodulin	2331	-4,21
down	SLC40A1	solute carrier family 40 (iron-regulated transporter), member 1	30061	-4,39
down	NFKBIZ	nuclear factor of kappa light polypeptide gene enhancer in B-cells inhibitor, zeta	64332	-4,39
down	BRINP1	bone morphogenetic protein/retinoic acid inducible neural-specific 1	1620	-4,41
down	TNXB	tenascin XB	7148	-4,51
down	DPT	dermatopontin	1805	-4,59

down	COLEC12	collectin sub-family member 12	81035	-4,82
down	SERPINF1	serpin peptidase inhibitor, clade F (alpha-2 antiplasmin, pigment epithelium derived factor), member 1	5176	-5,09
down	WISP2	WNT1 inducible signaling pathway protein 2	8839	-5,56
down	MME	membrane metallo-endopeptidase	4311	-5,74
down	ANGPTL2	angiopoietin-like 2	23452	-5,78
down	PCSK7	proprotein convertase subtilisin/kexin type 7	9159	-5,79
down	RSPO3	R-spondin 3	84870	-5,82
down	DKK2	dickkopf WNT signaling pathway inhibitor 2	27123	-6,02
down	PTGDS	prostaglandin D2 synthase 21kDa (brain)	5730	-6,09
down	TMTC1	transmembrane and tetratricopeptide repeat containing 1	83857	-6,40
down	RCAN2	regulator of calcineurin 2	10231	-6,64
down	APOD	apolipoprotein D	347	-7,50
down	FOSB	FBJ murine osteosarcoma viral oncogene homolog B	2354	-7,52
down	CTSK	cathepsin K	1513	-7,73
down	FOS	FBJ murine osteosarcoma viral oncogene homolog	2353	-7,77
down	MMP1	matrix metallopeptidase 1 (interstitial collagenase)	4312	-8,62
down	PDGFD	platelet derived growth factor D	80310	-10,27
down	ABCA6	ATP-binding cassette, sub-family A (ABC1), member 6	23460	-14,56
down	DPP4	dipeptidyl-peptidase 4	1803	-15,95
down	ABCA9	ATP-binding cassette, sub-family A (ABC1), member 9	10350	-17,06
